# An adipocentric perspective of pancreatic lipotoxicity in diabetes pathogenesis

**DOI:** 10.1530/JOE-23-0313

**Published:** 2024-05-15

**Authors:** Renata Risi, Antonio Vidal-Puig, Guillaume Bidault

**Affiliations:** 1 Department of Experimental Medicine, Sapienza University of Rome, Sapienza University of Rome, Rome, Italy; 2 University of Cambridge Metabolic Research Laboratories, Wellcome Trust-MRC Institute of Metabolic Science, Cambridge, UK; 3 Cambridge University Nanjing Centre of Technology and Innovation, Nanjing, P. R. China; 4 Centro de Investigacion Principe Felipe, Valencia, Spain

**Keywords:** adipokines, adipose tissue, diabetes, lipotoxicity, pancreas

## Abstract

Obesity and diabetes represent two increasing and invalidating public health issues that often coexist. It is acknowledged that fat mass excess predisposes to insulin resistance and type 2 diabetes mellitus (T2D), with the increasing incidence of the two diseases significantly associated. Moreover, emerging evidence suggests that obesity might also accelerate the appearance of type 1 diabetes (T1D), which is now a relatively frequent comorbidity in patients with obesity. It is a common clinical finding that not all patients with obesity will develop diabetes at the same level of adiposity, with gender, genetic, and ethnic factors playing an important role in defining the timing of diabetes appearance. The adipose tissue (AT) expandability hypothesis explains this paradigm, indicating that the individual capacity to appropriately store energy surplus in the form of fat within the AT determines and prevents the toxic deposition of lipids in other organs, such as the pancreas. Thus, we posit that when the maximal storing capacity of AT is exceeded, individuals will develop T2D. In this review, we provide insight into mechanisms by which the AT controls pancreas lipid content and homeostasis in case of obesity to offer an adipocentric perspective of pancreatic lipotoxicity in the pathogenesis of diabetes. Moreover, we suggest that improving AT function is a valid therapeutic approach to fighting obesity-associated complications including diabetes.

## Introduction

Obesity results from an imbalance between dietary intake and energy expenditure, leading to the storage of excess energy in the form of lipids within adipose tissue (AT). The increasing prevalence of obesity worldwide is closely associated with a subsequent rise in the incidence of diabetes (GBD 2021 Diabetes Collaborators 2023), making obesity a widely recognised risk factor for the development of type 2 diabetes mellitus (T2D) (Klein *et al.* 2022). Additionally, emerging evidence also indicates that obesity accelerates the development of type 1 diabetes mellitus (T1D) (March *et al.* 2021), representing a risk factor for health in these patients (Cantley *et al.* 2021). In this review, we summarise recent advances linking obesity to the development and control of diabetes, focussing on the interplay between the AT and the pancreas.

The close link between obesity and diabetes is supported by the evidence that increased body mass index (BMI) is associated with a higher risk for T2D (Colditz *et al.* 1995). Body fat accumulation, especially the android type of obesity where fat accumulates in visceral and abdominal sites, has been associated with systemic metabolic disturbances since 1947 when a link with insulin resistance (IR) was proposed (Vague 1947). Prospective studies with subjects at risk for T2D demonstrate that the development of abdominal obesity is correlated with the loss of β-cell function (Cnop *et al.* 2005, Menzaghi *et al.* 2006, De Boer *et al.* 2007). The first compensatory mechanism to IR is the expansion of β-cell mass and increased insulin secretion (Christensen & Gannon 2019). In addition, it has been proposed that excess adiposity could be the primary cause of initially increasing insulin secretion and IR (McGarry 2002, Fryk *et al.* 2021). Moreover, when this compensatory mechanism fails, β-cell loss and dysfunction occur, leading to hyperglycaemia and diabetes.

It is worth noting that the relationship between T2D and obesity is not linear, as not all obese people develop T2D at the same level of adiposity (Sims 2001). Evidence indicates that the individual capacity to appropriately store energy surplus in the form of fat within the AT determines and prevents toxic lipid deposition in other organs, such as the pancreas and liver (Virtue & Vidal-Puig 2010). Thus, the threshold when the maximal energy storage capacity within the AT is reached could be the individualised critical point triggering T2D onset. Therefore, we posit that understanding the factors regulating AT dysfunction and failure is essential in identifying new therapeutic targets to prevent and delay insulin secretion disruption. Here, we review the mechanisms connecting the AT expansion, dysfunction and failure linked to increased lipid influx towards the pancreas, the effects of lipid regulation on insulin secretion and the mechanism of lipotoxicity in β cells.

### Disconnecting BMI from diabetes: the adipose tissue expansion failure?

From an epidemiological perspective, BMI, IR and T2D are undeniably connected. However, BMI is a crude measure that does not fully grasp the complexity of the link between excessive fat accumulation and IR, compensatory increased insulin secretion, and ultimately, β-cell failure. The complexity of this relationship is evidenced by the fact that obesity and T2D do not associate similarly in some subpopulations (Sims 2001). For example, men develop T2D at a lower BMI than women (Logue *et al.* 2011), and metabolic impairments occur in the Asian population at a lower average BMI than in the Caucasian population (Barnes 2011).

One mechanism that could explain these controversies is the AT expandability hypothesis. According to this hypothesis, every individual possesses an intrinsic capacity to expand their AT in positive energy balance. When the AT storage capacity is exceeded, the physiological function of AT to buffer lipid flux is blunted (Virtue & Vidal-Puig 2010), with a reduction of anti-lipolytic response to insulin in the postprandial states and a failure to take up lipids from the circulation (Morigny *et al.* 2021). In states of AT dysfunction, excess energy in the form of lipids is redirected towards non-adipose organs such as the liver, the skeletal muscle, the heart or the pancreas. Ectopic lipid accumulation in non-adipose organs causes organ dysfunction, often called lipotoxicity (Virtue & Vidal-Puig 2010). Moreover, overwhelmed adipocytes also express and secrete higher levels of proinflammatory adipokines and chemoattractants, promoting an inflamed state of the AT and systemic inflammation that further impedes insulin sensitivity (Hotamisligil 2017).

Individuals with congenital or acquired defects in AT, known as lipodystrophic syndromes, demonstrate the most severe manifestation of limited AT expansion (Garg 2011). In lipodystrophic syndromes, the loss or dysfunction of AT leads to lipid storage in ectopic sites, and thus to IR, diabetes, dyslipidaemia and liver complications (Vatier *et al.* 2013). The metabolic characterisation of three patients with one rare lipodystrophic syndrome, namely neutral lipid storage disease with myopathy, due to homozygosity for loss-of-function mutations in the *ATGL* gene, highlighted the consistent significant increase in pancreatic fat and visceral fat in these conditions, with subsequent impaired insulin response to glucose (Natali *et al.* 2013).The relationship between lipodystrophy and diabetes underscores the crucial role of AT homeostasis in maintaining metabolic health. A proof of concept for this concept has been demonstrated through fat depot transplantation into the lipoatrophic mouse model A-Zip, effectively reversing the metabolic defects induced by lipoatrophy (Gavrilova *et al.* 2000). Notably, the transplantation of leptin-deficient fat pads into the A-Zip mouse does not rescue the metabolic defects (Gavrilova *et al.* 2000), implying that leptin deficiency plays a crucial role in mediating lipoatrophy-induced metabolic dysfunction. Following this study, administering leptin to patients with genetic or acquired, partial or generalised lipodystrophy with decreased serum leptin levels can reverse excessive hyperphagia and improve metabolic irregularities, notably restoring insulin secretion and sensitivity (Vatier *et al.* 2016). While complex, the link between AT dysfunction and the development of the cardiometabolic complications of obesity, such as diabetes, is established, and the fluxes of lipids towards non-adipose organs could explain the aetiology of metabolic dysfunctions.

### Lipids in the pancreas: the double-edged sword

Diabetes and dyslipidaemia are interconnected conditions that often coexist and contribute to each other's progression. Increased serum free fatty acid (FFA) levels, notably in their saturated form, are positively associated with the incidence of T2D (Wang *et al.* 2003, Qureshi *et al.* 2019). Abnormal intracellular lipid accumulation and adipocyte infiltration in the pancreas, generally defined as ‘pancreatic steatosis’, is a pathological event associated with obesity and metabolic syndrome that predisposes to β-cell dysfunction and death (Smits & Van Geenen 2011). On the other hand, fatty acids (FAs) also promote insulin secretion, which suggests they might be initial signals communicating the need for increased insulin secretion to blunt lipolysis. These observations highlight the dual role of FAs in regulating β-cell function and the necessity to maintain appropriate lipid homeostasis to ensure appropriate glucose control.

### Lipid regulation of insulin secretion

Lipids have critical physiological actions in pancreatic β cells, acting as a cofactor to boost glucose-stimulated insulin secretion (GSIS). Accordingly, a minimal level of FAs is essential for normal GSIS. Islets deprived of FAs lose their GSIS ability, which can be rescued by supplementing exogenous FAs (Stein *et al.* 1996, Roduit *et al.* 2004). Homeostatically, the increased serum FAs observed in insulin-resistant individuals might be an adaptation to potentiate insulin secretion and compensate for increased insulin needed to compensate for IR (McGarry 2002, Prentki *et al.* 2002, Nolan *et al.* 2006). Interestingly, the insulinotropic action of FAs may be linked to their chain length and degree of saturation. It has been shown that saturated FAs (SFAs), such as palmitic (C16:0) and stearate (C18:0), are better enhancers of GSIS than mono- and polyunsaturated FAs in the perfused rat pancreas (Stein *et al.* 1997). The converse has been observed in human islets where palmitoleate and oleate induced insulin secretion more potently than their saturated counterparts, palmitate and stearate (Cen *et al.* 2016). Others showed that increasing FFAs failed to potentiate insulin secretion in response to mixed meals and to intravenous glucose in non-diabetic subjects without a family history of diabetes, suggesting that genetic predisposition also plays an essential role in lipid regulation of GSIS (Kashyap *et al.* 2003). On the other hand, it should be highlighted that the relation between FFAs levels and insulin secretion is not linear. In fact, sustained lowering FAs levels achieved with long-term therapy Acipimox was shown to increase insulin secretion rates in a subset of patients with diabetes (Davoren *et al.* 1998) or at risk of diabetes (Paolisso *et al.* 1998), suggesting that FFAs homeostasis is important for insulin secretion regulation, especially in people at risk.

The precise mechanisms by which FAs enhance GSIS are partially unknown but seem to act at the extra- and intracellular levels (Prentki *et al.* 2013). We summarised them in Fig. 2A. Pancreatic β cells can sense extracellular FA through the free fatty acid receptor 1 (FFAR1) (Itoh *et al.* 2003). FFAR is a G-protein receptor highly expressed in β cells and responsible for mediating approximately 50% of the FA-induced insulin secretion (Prentki *et al.* 2013). Accordingly, high-fat diet (HFD)-fed mice lacking FFAR1 (GPR40) show a reduction of insulin secretion during i.v. glucose tolerance test when compared to WT littermates (Kebede *et al.* 2008). Significantly, FFAR1 activation does not trigger insulin secretion in the absence of glucose. Therefore, it has been hypothesised that FFAR1 agonists could improve insulin secretion without increasing the risk of hypoglycaemia in patients with T2D (Kebede *et al.* 2008). To note, FFAR1 recently gained attention as a potential pharmacological target in T2D (Li *et al.* 2018). However, research on FFAR1 agonists did not result in any available drugs because of hepatotoxicity due to the lipophilic nature of the agonists rather than their direct receptor-mediated action (Kaku *et al.* 2016). β cells also express FFAR2 and FFAR3 receptors, which are activated by short-chain FAs (under six carbons), mainly produced in the colon by gut microbiota when abundant undigested carbohydrates exist (Mishra *et al.* 2020). However, the role of these receptors regulating insulin release from β cells appears to be non-significant (Ichimura *et al.* 2014).

The metabolism of intracellular lipids is closely connected to glucose metabolism. Additionally, the esterification and lipolysis of neutral lipids, also known as glycerolipids/fatty acid cycling, play a supportive role in GSIS in β cells. Fatty acid uptake is a crucial initial step in regulating GSIS through intracellular targets. Unlike glucose, FAs easily cross the phospholipid bilayer due to their low energy barrier (Hamilton & Kamp 1999), facilitated by proteins like CD36 and fatty acid transport protein (FATP) on the plasma membrane (Noushmehr *et al.* 2005). Importantly, β cells release FAs from the plasma membrane, a process dependent on glucose concentration, to activate the FFAR1 receptor and promote GSIS (Mugabo *et al.* 2017). Interference with FA release leads to reduced calcium oscillation and impaired insulin release in β cells (Mugabo *et al.* 2017).

Lipolysis of intracellular TAGs generates FAs, which are activated to LC-CoA (long-chain FA-CoA) by long-chain acyl-CoA synthase (ACSL), before undergoing β-oxidation. The increase of intracellular glucose concentration increases the generation of malonyl CoA, suppresses the carnitine palmitoyl transferase 1 (CPT1) activity and reduces β-oxidation (Cantley *et al.* 2019). Inhibition of β-oxidation resulted in increased availability of LC-CoA that promotes GSIS by targeting ATP-sensitive potassium channels and protein kinase C (Larsson *et al.* 1996, Yaney *et al.* 2000) and by entering the glycolipid (GL)/FFA cycle (Prentki & Madiraju 2012). The GL/FFA cycle is a process of FA esterification with glycerol to form diacylglycerol (DAG) and triacylglycerols (TAGs) (lipogenic phase), followed by the hydrolysis of TAGs to regenerate monoacylglycerol (MAG), glycerol and FAs (lipolytic phase), which can re-enter the cycle. FFAs generated can exit the cell and activate FFAR1, while MAGs directly bind and activate Munc13-1, an insulin granule exocytosis-facilitating protein (Prentki & Madiraju 2012).

In addition, lipid droplets (LDs) are recognised to play an integral part in the GL/FFA cycle. LDs are intracellular organelles consisting of a monolayer phospholipid membrane and a lipid core composed of TAGs, cholesterol ester and retinol ester (Welte & Gould 2017) that are detected in adult islet β cells (Vernier *et al.* 2012, Trevino *et al.* 2015, Dai *et al.* 2016). LD formation starts as TAG accumulation within the endoplasmic reticulum (ER) membrane bilayer, and newly synthesised TAGs are primarily transferred to LDs (Nettebrock & Bohnert 2020). After emerging from the ER, LDs can grow by directly incorporating FAs into TGs via DGAT2 (Gluchowski *et al.* 2017). LD degradation releases MAG and FFAs, which support GSIS as previously described. Lipolysis of LDs is initiated by adipose triglyceride lipase (ATGL) and is regulated by multiple factors, including phosphorylation of PLIN1/5, recruitment and activation of lipases to LDs (Sztalryd & Brasaemle 2017, Yang *et al.* 2023). Two models of β-cell-specific ATGL knockout mice proposed that ATGL deficiency blunts insulin secretion (Tang *et al.* 2013, Attané *et al.* 2016). Moreover, LDs in pancreatic islets accumulate in cases of nutritional stress, diabetes and dysfunction of β cells (Tong *et al.* 2020), suggesting that impaired LD production and degradation might be involved in impaired insulin secretion.

To sum up, lipids and their metabolism are critical regulators of insulin secretion but act as potentiators more than inducers. Accordingly, FAs cannot trigger insulin secretion in the absence of glucose. However, excess lipids in the pancreas can alter β-cell functions, as discussed in more detail in the section ‘Lipotoxicity in pancreas’.

### Lipid regulation of glucagon secretion

Glucagon is a peptidic hormone secreted by the α cells of the pancreas when glucose availability is low, promoting gluconeogenesis and lipolysis in the liver and preventing hypoglycaemia and fuel availability. While glucagon has been demonstrated to induce lipolysis in isolated adipocytes, the expression of the glucagon receptor within adipocytes is significantly lower compared to that in hepatocytes (Duncan *et al.* 2007). Furthermore, glucagon concentration in the peripheral circulation is notably lower than in the portal vein (Duncan *et al.* 2007). Together with insulin, it regulates whole-body glucose homeostasis. In advanced stages of T2D and autoimmune diabetes, pancreatic α cells undergo deregulation, losing the ability to respond to hypoglycaemia appropriately. Clinically, this results in life-threatening hypoglycaemia, which is a limiting factor in diabetes management in clinical setting (Panzer and Caicedo 2021).

Plasmatic low glucose levels are the main signal triggering glucagon secretion in rodent and human islets (Ohneda *et al.* 1969). Interestingly, a recent study highlighted that fatty acid oxidation is required for the inhibitory effect of glucose on glucagon secretion by reducing ATP levels (Armour *et al.* 2023). On the other hand, FAs are known to promote glucagon secretion. Most studies show that short-term exposure to FAs stimulates glucagon release in isolated islets and clonal α-cell lines (Bollheimer *et al.* 2004, Olofsson *et al.* 2004). Short-term treatment with the saturated FA palmitate increases glucagon secretion by enhancing α-cell intracellular calcium entry and by relieving the inhibitory paracrine action of somatostatin, which is secreted from pancreatic δ cells (Olofsson *et al.* 2004). Similarly, the polyunsaturated FA linoleic acid increases glucagon secretion in isolated mouse and rat islets and in In-R1-G9 glucagonoma cells (Wang *et al.* 2011). Accordingly, the long-term culture of α cells with palmitate augmented glucagon release and enhanced glucagon expression and protein content, probably by activating the mitogenic mitogen-activated protein kinase (MAPK) pathway (Piro *et al.* 2010). Similarly to FA-induced insulin secretion, FA-induced glucagon secretion could also be mediated by FFAR1, constitutively expressed on the surface of α cells (Flodgren *et al.* 2007, Wang *et al.* 2011).

Similarly to β cells, FAs can also impair glucagon secretion. The inhibitory action of insulin on glucagon release was impaired when α cells were incubated with FA for a prolonged period of time. This effect was attributed to palmitate-induced insulin resistance on the insulin receptor substrate 1 (IRS-1)/phosphatidylinositol kinase (PI3K)/serine-threonine protein kinase (Akt) pathway (Piro *et al.* 2010). Of note, the survival of α cells, like β cells, is affected by glucolipotoxic conditions, as high palmitate combined with high glucose levels are able to induce apoptosis in rodent α cells (Piro *et al.* 2010).

In general, all these data support the hypothesis that α-cell secretory capacity is regulated by lipid signaling. In addition, α cells are susceptible to lipotoxicity, which contributes to the loss of the counterregulatory effect of glucagon in glycaemic control in patients with diabetes.

### Lipotoxicity in pancreas

While a minimal level of FAs is essential for normal glucose-dependent insulin secretion, prolonged exposure of islets to increased levels of FAs is associated with impaired β-cell function, leading to reduced capacity to secrete sufficient insulin to counteract IR. The detrimental effects of chronic lipid overload in pancreatic islets are well-established. It was discovered decades ago that exposure of islets isolated from Zucker rats to lipotoxic doses of FA (oleate and palmitate) results in ceramide-induced cell death (Shimabukuro *et al.* 1998*a*,*b*). One of the first *in vivo* evidence linking dyslipidaemia with impaired β-cell function was observed in hyperglycaemic Goto-Kakizaki rats, where the hyperlipidaemia induced by high-fat feeding markedly impaired glucose-induced insulin secretion in this model (Briaud *et al.* 2002).

Exposure to chronically high levels of lipids leads to cell injury through lipotoxic mechanisms (Fig. 2B). The mechanisms driving lipotoxicity in β cells are still to be fully explored but encompass signaling through FA receptors, ceramide production, and cell stress responses such as ER stress and mitochondrial dysfunction, as recently reviewed here (Oh *et al.* 2018).

Ceramide accumulation represents one of the primary mechanisms underlying lipotoxicity in β cells. Long-chain saturated FAs, such as palmitate, stearate and arachidate, stimulate *de novo* ceramide synthesis and formation (Chavez & Summers 2003). Ceramides induce apoptosis in β cells by induction of ER stress, alterations of mitochondrial membrane integrity and inhibition of the pro-survival kinase Akt (García-Ruiz *et al.* 2002, Chavez *et al.* 2003, Véret *et al.* 2011, Boslem *et al.* 2013). Accordingly, overexpression of CerS4 potentiates palmitate-induced accumulation of ceramides and enhances lipotoxicity in β cells (Véret *et al.* 2011). Similarly, exposure of β cells to the ceramide analogue C_2_-ceramide enhances the pro-apoptotic and anti-proliferative effect of palmitate (Maedler *et al.* 2003). Conversely, inhibition of *de novo* ceramide synthesis using serine palmitoyl transferase (l-cycloserine) or ceramide synthase (fumonisin-B1) inhibitors attenuates FA-induced β-cell apoptosis and lowers hyperglycaemia in obese Zucker diabetic fatty rats *in vivo* (Shimabukuro *et al.* 1998*a*,*b*). Interestingly, β cells defend themselves from ceramide-induced dysfunction by secreting an active neutral ceramidase to lower FA-induced apoptosis (Tang *et al.* 2017).

ER stress is also an essential mediator of the lipotoxic insults. *In vitro* and *in vivo* studies have shown that β cells activate the unfolded protein response (UPR) following glucolipotoxic insults to maintain ER function and survival (Eizirik *et al.* 2008, Shrestha *et al.* 2020). However, excessive and prolonged UPR activation leads to the initiation of apoptosis. Both high levels of glucose (Elouil *et al.* 2007) and FAs, mainly when saturated (Cunha *et al.* 2008, Marselli *et al.* 2020), are associated with increased ER stress in human islets. In particular, SFAs induce sustained activation of the three arms of the UPR response IRE1, PERK and ATF6 (Cnop *et al.* 2007, Cunha *et al.* 2008), resulting in β-cell apoptosis. Multiple mechanisms by which SFAs directly or indirectly impair cell function through ER stress have been proposed, including protein palmitoylation (Baldwin *et al.* 2012) and impairment of sphingolipid metabolism (Boslem *et al.* 2011, Véret *et al.* 2013). These defects most likely act synergically, leading to β-cell dysfunction and apoptosis.

Altogether, there is compelling evidence that high levels of FAs, particularly their saturated counterparts, can induce a lipotoxic response evidenced by increased ceramide production and ER stress leading to β-cell dysfunction and apoptosis. These data indicate a direct role for lipotoxicity in decreasing β-cell mass in obesity-induced dyslipidaemia. Efforts to limit the lipid fluxes towards the pancreas appear promising in preventing β-cell mass reduction in obesity, thus preventing T2D onset, in addition to appropriate management of peripheral IR.

### Adipose tissue control of pancreatic lipid content and function

Ogilvie *et al.* were the first to observe that obesity is associated with hypertrophy of the islets of Langerhans in the pancreas (Ogilvie 1933). Later on, Olsen *et al.* associated pancreatic steatosis with a decreased ability to secrete insulin and the development of T2D (Olsen 1978). Since then, fat accumulation in the pancreas has been referred to with various synonyms, such as pancreatic lipomatosis, fatty replacement, fatty pancreas, non-alcoholic fatty pancreatic disease or pancreatic steatosis (Smits & Van Geenen 2011). This nomenclature has the limitation of not distinguishing between the accumulation of triglycerides in acinar cells, β cells or intrapancreatic adipocytes. Histological observations of HFD-induced pancreatic steatosis showed that pancreatic ectopic fat is characterised by adipocyte infiltration rather than intracellular fat accumulation (Pinnick *et al.* 2008). Nonetheless, electron microscopy has also visualised intracellular fat accumulation even earlier than ectopic fat deposition in the pancreas itself (Moffitt *et al.* 2005, Yan *et al.* 2006).

The metabolic consequences of pancreatic ectopic adipocyte infiltration have been recently reviewed by Wagner *et al.* (2022). While preclinical evidence showed that physiologically pancreatic adipocytes stimulate insulin secretion in β cells by releasing FAs (Quiclet *et al.* 2019), clinical studies found that the pathological development of fatty pancreas is linked to β-cell dysfunction and reduced insulin secretion (Tushuizen *et al.* 2007, Van Der Zijl *et al.* 2011, Chin *et al.* 2021, Yamazaki *et al.* 2022), although to what extent this association is preserved when diabetes is overt is uncertain (Tushuizen *et al.* 2007, Lu *et al.* 2019, Chin *et al.* 2021).

### Regulation of lipid accumulation in the pancreas

The development of pancreatic lipid accumulation results from an imbalance between lipid synthesis, uptake and disposal. While *de novo* lipogenesis does not substantially contribute to pancreatic islets’ fatty acid content, at least in non-stimulated states, circulating FFAs represent the primary source of pancreatic lipids. Physiologically, FFA serum levels increase during the fasted state due to AT lipolysis (Kersten 2023). Pathologically, obesity is associated with an increased FFA availability that pancreatic cells can take up (Fryk *et al.* 2021). FA uptake is mediated by specific transport membrane proteins, FA translocase (CD36), FA binding proteins and FATPs (Pownall & Moore 2014). It has been shown that CD36 is responsible for FA uptake and lipid accumulation in β cells (Khan 2018). CD36 expression is upregulated in the obese diabetic population, and inhibition of its activity *in vitro* enhances insulin secretion (Nagao *et al.* 2020). Following uptake through CD36, the FA is then activated by FATPs, which are acyl-coenzyme A (CoA) synthases that add a CoA residue to increase their water solubility (Pownall & Moore 2014). However, despite being partly regulated by transporters, the uptake of FFA by cells is primarily determined by the concentration gradient of FAs across the plasma membrane (Hamilton & Kamp 1999). The increase in the extracellular concentration of FFAs and ‘metabolic trapping’ of FA within cells through the formation of acyl-CoA (FATP) all promote cellular fatty acid uptake (Pownall & Moore 2014).

Beside circulating FFA, β cells can uptake lipids from lipoproteins through the action of lipoprotein lipase (LPL), and this process increases with aging (Cnop *et al.* 2000). However, to our knowledge, there has been no concrete evidence so far supporting the notion that the export of very low-density lipoprotein (VLDL)-TAG is the primary source of fat accumulation in the pancreas. However, it has been demonstrated that diabetes remission is linked to decreased hepatic VLDL-TG export, while a return to a diabetic state is associated with increased plasma VLDL-TAG levels (Al-Mrabeh *et al.* 2020). Surprisingly, murine-specific deletion of LPL in β cells does not reduce pancreatic fat and LPL overexpression in β cells only results in a mild increase in islet TAG content. However, deletion or overexpression of LPL in β cells profoundly reduces insulin secretion (Pappan *et al.* 2005), which seems independent of pancreatic lipid content.

Altogether, it can be assumed that the bioavailability of serum lipids appears to be the main driver regulating FA uptake in the pancreas.

### Regulation of lipid availability by the AT

One critical organ regulating the availability of lipids is the AT. The lipid fluxes to the pancreas in fasting and fed states heavily rely on AT, which is the major contributor to plasma FFA concentrations. Various dysregulated processes in obese AT can potentially impact the transport and bioavailability of FFAs in the pancreas. The primary function of AT is energy storage and release in the form of fat, acting as a ‘buffer’ to the influx of dietary fat (Frayn 2002). White adipocytes are characterised by their large unilocular central lipid droplet, where fat is stored as neutral TAGs and released in the form of FFAs, depending on the energy status.

In particular, in conditions of increased energy demand such as fasting and exercise, FA mobilisation and release in the bloodstream are promoted by reduced plasma insulin and increased release of adrenaline and noradrenaline via the activity of lipases (Gjedsted *et al.* 2007). Conversely, following meal ingestion, the postprandial increase in plasma insulin efficiently blocks lipid export by suppressing lipolysis and promotes the storage of dietary lipids within the AT (Sadur & Eckel 1982, Roust & Jensen 1993). In particular, in the fed state, adipocytes take up and store lipids from the circulation in the form of FAs released from circulating triglyceride-containing lipoproteins such as chylomicrons and VLDLs bound to the capillary endothelium through the activity of LPL (Sniderman 2000). In 1996, Binnert *et al.* showed a rapid appearance in the plasma of labelled FAs after ingestion of labelled fat in humans, suggesting that there is an entry of FAs deriving from exogenous TAGs in serum FFAs pool (Binnert *et al.* 1996). Similarly, Fielding *et al.* showed that dietary fat contributes to specific post-prandial FAs profile (Fielding *et al.* 1996). This evidence together suggests that the AT does not take up a quote of FFAs arising from the hydrolysis of TAGs by LPL and joins the plasma pool in a process often referred to as ‘spillover’ (Karpe *et al.* 2011). It is believed that the spillover of FFAs rate is higher for chylomicrons, at approximately 30%, (Pichè *et al.* 2018), compared to VLDL, whose contribution to the FFAs pool is negligible (Bush *et al.* 2014). Intriguingly, the spillover of chylomicrons into the bloodstream appears to be more significant in lean individuals and in women but does not seem to be a pathway providing excess FFAs to non-adipose organs in obesity (Piché *et al.* 2018). This observation casts doubt on the contribution of FFAs spillover from the AT to the bloodstream FFAs pool in metabolic disease. It is worth considering that lipoprotein hydrolysis of both chylomicrons and VLDL may not be complete under specific conditions, leading to the formation of lipoprotein remnants. Interestingly, higher levels of total, very large, large, and very small (therefore reflecting remnant lipoprotein) TAG-rich lipoproteins are typically present at high concentrations in patients with T2D and are associated with cardiovascular diseases (Ginsberg *et al.* 2021, Matsushima-Nagata *et al.* 2023).

The release of FAs from upper-body subcutaneous AT significantly determines systemic FFAs levels (Nielsen *et al.* 2004). However, the association between obesity, FFAs serum levels and IR is not as linear as expected. It has been described that plasma FFAs levels are associated with obesity (Arner & Rydén 2015), increased fat mass (Boden 2008) and T2DM (Fraze *et al.* 1985). Nonetheless, greater postprandial FFA excursions in blood have also been associated with T2D and obesity compared to non-diabetic controls (Miles *et al.* 2003). Hence, raised postprandial FFA levels have been proposed as a pathogenetic hallmark leading to the development of β-cell dysfunction (Carpentier 2008). However, as reviewed by Karpe *et al.* in 2011, the association between obesity and increased FFA levels observed by most of the studies is relatively marginal and not consistent (Karpe *et al.* 2011). Moreover, as the fat mass increases, FFA release per kilogram of fat mass is downregulated, rather than increased, which might explain why several patients with obesity display normal FFAs serum concentration despite the excessive fat (Karpe *et al.* 2011). In line with this, McQuaid *et al.* found that men with obesity had normal systemic fasting FFAs concentration compared to lean control, but downregulation of FFAs delivery to the AT and decreased AT storage capacity after meal (McQuaid *et al.* 2011).

All these observations highlight that AT regulation and dysregulation in the case of obesity and IR are complex and still to be fully understood. However, AT is a crucial regulator of lipid bioavailability and metabolism, making AT an essential regulator of lipid deposition in the pancreas. AT dysfunction in obesity results in insufficient suppression of lipolysis and impaired lipid storage capacity, altogether contributing to the increased pancreatic FA influx of obese individuals.

### Not all fat is bad

Body fat distribution, more than total body fat, is associated with the risk for glucose metabolism disruption. Classically, AT is divided into subcutaneous white AT (scWAT), which physiologically constitutes about 80% of all body fat, and visceral white AT (vWAT), which accounts for up to 10–20% of total fat in men and 5–8% in women. It increases with age (Ibrahim 2010). scWAT is located predominantly in the femorogluteal regions, while vWAT is in the abdominal cavity around internal organs. These two main body fat depots show significant endocrinological and metabolic differences.

Examination of WAT depots uncovered distinctive features between visceral and subcutaneous ATs. Notably, vWAT displays greater vascularity compared to scWAT, as observed by Villaret *et al.* (2010). Despite this enhanced vascularisation, vWAT exhibits a lower capacity for new vascular sprouting (Gealekman *et al.* 2011). Additionally, vWAT is characterised by increased innervation, a higher presence of inflammatory and immune cells, and a larger proportion of large adipocytes. Furthermore, vWAT pre-adipocytes demonstrate diminished differentiation potential, leading to an elevated percentage of large adipocytes (Skurk *et al.* 2007). vWAT cells exhibit heightened sensitivity to adrenergic stimulation and increased lipolysis (Arner *et al.* 1990, Hellmér *et al.* 1992, Lafontan & Langin 2009). Additionally, vWAT adipocytes are more prone to developing IR (Frayn 2000). Conversely, subcutaneous adipocytes in healthy individuals exhibit a greater propensity for the uptake of triglycerides and FFAs from the circulation, notably postprandially (Mårin *et al.* 1992, Misra & Vikram 2003) It therefore appears that scWAT has a higher storage capacity than vWAT. Besides their capacity to store lipids, the quality of fat stored differs between subcutaneous and visceral depots. Within obese subjects, vWAT is characterised by increased levels of non-essential MUFAs, likely *de novo* lipogenesis end products, and lower levels of the w6 arachidonic acid and w3 FAs such as DPA and DHA when compared to subcutaneous depots (Yew Tan *et al.* 2016,Petrus *et al.* 2017). However, no differences in FA composition were observed between metabolically healthy and unhealthy obese individuals, suggesting that the relative proportion of the depot sizes rather than a change in their intrinsic FA composition is associated with the development of IR (Yew Tan *et al.* 2016).

The secretion of adipokines and cytokines also differs between white adipose tissue depots. scWAT is characterised by a higher release of adiponectin (Drolet *et al.* 2009) and leptin (Van Harmelen *et al.* 1998). On the other hand, vWAT tends to release larger amounts of pro-inflammatory cytokines, such as IL-6 (Fontana *et al.* 2007). Concerning obesity, an increase of one standard deviation in subcutaneous fat mass leads to a 48% reduction in the likelihood of IR, while a one standard deviation increase in vWAT mass results in an 80% increase in the likelihood of IR (McLaughlin *et al.* 2011). In the Jackson Heart Study, while both abdominal vWAT and scWAT volumes were positively correlated with fasting plasma glucose and triglyceride levels, vWAT deposition in the abdomen was most strongly associated with hypertension, T2D, and metabolic syndrome risk (Liu *et al.* 2010). In line with this, Yamazaki *et al.* found that specific body fat distribution phenotype, particularly with the accumulation of AT in the liver, pancreas and the trunk, was associated with an increased incidence of T2D over 6 years of follow-up (Yamazaki *et al.* 2022)

All these data indicate that body fat distribution plays a significant role in diabetes pathogenesis, with upper body fat being closely associated with T2D development.

### Revisiting the non-lipid-mediated crosstalk between the AT and the pancreas

Besides regulating energy storage and release, AT also serves as an endocrine organ releasing pro- and anti-inflammatory adipokines, growth factors, cytokines, chemokines, and miRNAs, also called RNAkines (Ahima & Lazar 2008, Li *et al.* 2024). The secretion pattern of adipokines is dependent on body fat distribution and mass, with obesity being associated with increased production of proinflammatory mediators, such as TNF-alfa, leptin, IL-6, IL-12, IL-18 and the chemokines IL-8 and CCL2/MCP-1, rather than anti-inflammatory adipokines like adiponectin or omentin (Mancuso 2016). Adipokines and inflammation contribute to the crosstalk between AT and pancreatic β cells.

Amongst the adipokines mainly secreted by the adipocytes, leptin and resistin have been associated with impaired β-cell function and T2D, while adiponectin and omentin show beneficial effects. After the clinical evidence suggesting a relationship between circulating leptin levels and islet function (Ahima & Lazar 2008), leptin was the first adipokine associated with direct pancreatic effects. Leptin is a 16-kDa hormone secreted mainly by the adipocytes in the AT (Zhang *et al.* 1994) that regulates food intake at the level of the hypothalamus to maintain body fat stores (Klok *et al.* 2006). In obesity, leptin levels rise substantially as fat mass increases and in the context of leptin resistance, which limits its anorexigenic effects (Klok *et al.* 2006). Some peripheral organs express the leptin receptor, including β cells (Kieffer *et al.* 1996), and its activation triggers the MAPK/ERK pathway (Tanabe *et al.* 1997). High leptin levels have detrimental effects on β cells, inhibiting insulin secretion *in vitro* and *in vivo* and suppressing pre-proinsulin gene expression, further limiting insulin availability (Ahrèn & Larsson 1997, Seufert *et al.* 1999).

Resistin, also known as FIZZ3 or adipose tissue-specific secretory factor (ADSF), is a cysteine-rich polypeptide encoded by the RETN gene and mainly secreted by the adipocytes and to a lower level by leucocytes (Jamaluddin *et al.* 2012). It is believed to have a significant role in developing IR (Heilbronn *et al.* 2004, Menzaghi *et al.* 2006). A recent meta-analysis found that resistin levels were positively correlated with IR in T2D and obese individuals, suggesting its contribution in driving diabetes development (Su *et al.* 2019). Resistin reduces GSIS in pancreatic islets and INS-1E cell lines, but not in BTC-6 or BRIN-BD11 cells (Brown *et al.* 2007, Nakata *et al.* 2007, Sassek *et al.* 2016). It has been proposed that resistin renders β cells insulin resistant, likely through increased SOCS-3 expression and decreased Akt phosphorylation (Nakata *et al.* 2007, Sassek *et al.* 2016). However, resistin is also secreted by pancreatic islet cells, and its expression increases in diabetic patients (Al-Salam *et al.* 2011). Therefore, the contribution of AT-derived resistin in mediating β-cells dysfunction in obesity remains to be validated.

In contrast to leptin or resistin, adiponectin is a unique adipokine whose expression and circulating levels are inversely proportional to adiposity levels (Cnop *et al.* 2003, Ryan & Li 2022). The insulin sensitivity-promoting properties of adiponectin are well-established. Accordingly, adiponectin-deficient mice develop IR (Maeda *et al.* 2002), while adiponectin-overexpressing mice are protected against IR despite being more obese than their littermate controls (Kim *et al.* 2007). Adiponectin's beneficial actions are partially mediated by its capacity to promote healthy recruitment of new adipocytes (i.e. hyperplasia), increasing lipid buffering capacity in the context of obesity (Kim *et al.* 2007). Adiponectin also has direct beneficial effects on pancreas. Adiponectin receptors are expressed in the pancreas, and adiponectin mitigates β-cell loss by neutralising inflammatory and lipotoxic ceramides and diacylglycerols (Ye *et al.* 2015). Regarding omentin, a meta-analysis showed that lowered omentin-1 levels could serve as a biomarker for gestational diabetes mellitus and type 2 diabetes mellitus. However, additional investigations are needed to validate the role of omentin in preventing T2D (Pan *et al.* 2019).

Chronic inflammation of the AT of people with obesity is a well-known hallmark of metabolic disease. Immune dysregulation in AT is characterised by increased infiltration and activation of innate and adaptive immune cells, particularly AT macrophages, constituting up to 40% of all AT cells in obesity. Metabolically-activated AT macrophages are polarised into a pseudo-proinflammatory phenotype, secreting proinflammatory cytokines, such as TNF-α, IL-5, IL-1 and NO, capable of impairing insulin signaling in the AT and indirectly predisposing to diabetes (Kratz *et al.* 2014, Russo & Lumeng 2018). Interestingly, *in vitro* evidence also highlighted the direct detrimental effects of TNF-α on pancreatic-mediated insulin release (Zhang & Kim 1995, Sato *et al.* 2018).

Some obesity-related AT-derived miRNAs are also reported as emerging regulators of pancreatic β-cell function. For example, miR-132, whose levels are decreased in both AT and circulation of people with obesity (Heneghan *et al.* 2011), is beneficial for β-cell proliferation (Mziaut *et al.* 2020). In addition, serum levels of miR-15b and miR-146b were increased in children with obesity and adults with T2D, and overexpression of miR-15b and miR-146b in pancreatic β-cell line MIN6 cells decreased insulin secretion (Cui *et al.* 2018).

AT also secretes extracellular vesicles (EVs). These membrane-bound particles act as vehicles communicating organs and tissues, delivering proteins, mRNAs or miRNAs (Huang-Doran *et al.* 2017) It has been shown that EVs isolated from inflamed rodent and human adipocytes exert detrimental effects on pancreatic islets, survival and function, suggesting that EVs represent essential mediators in the AT-pancreas crosstalk (Gesmundo *et al.* 2021).

Finally, recent evidence suggests that basal insulin secretion may not depend solely on glucose levels but also on specific AT signals. In fact, stimulation of β3-adrenergic receptor in the AT induces insulin secretion while reducing blood glucose levels (Grujic *et al.* 1997). Similarly, a preprint recently demonstrated that acute AT-specific inhibition of PI3K activity leads to an increased insulin secretion without increasing glycaemia (Becattini *et al.* 2023). Both models are associated with increased FFA levels. Considering that FFAs alone does not trigger insulin secretion, this data suggests that the AT releases an incretin signal, yet to be discovered, to enhance insulin secretion in the fasted state.

**Figure 1:**
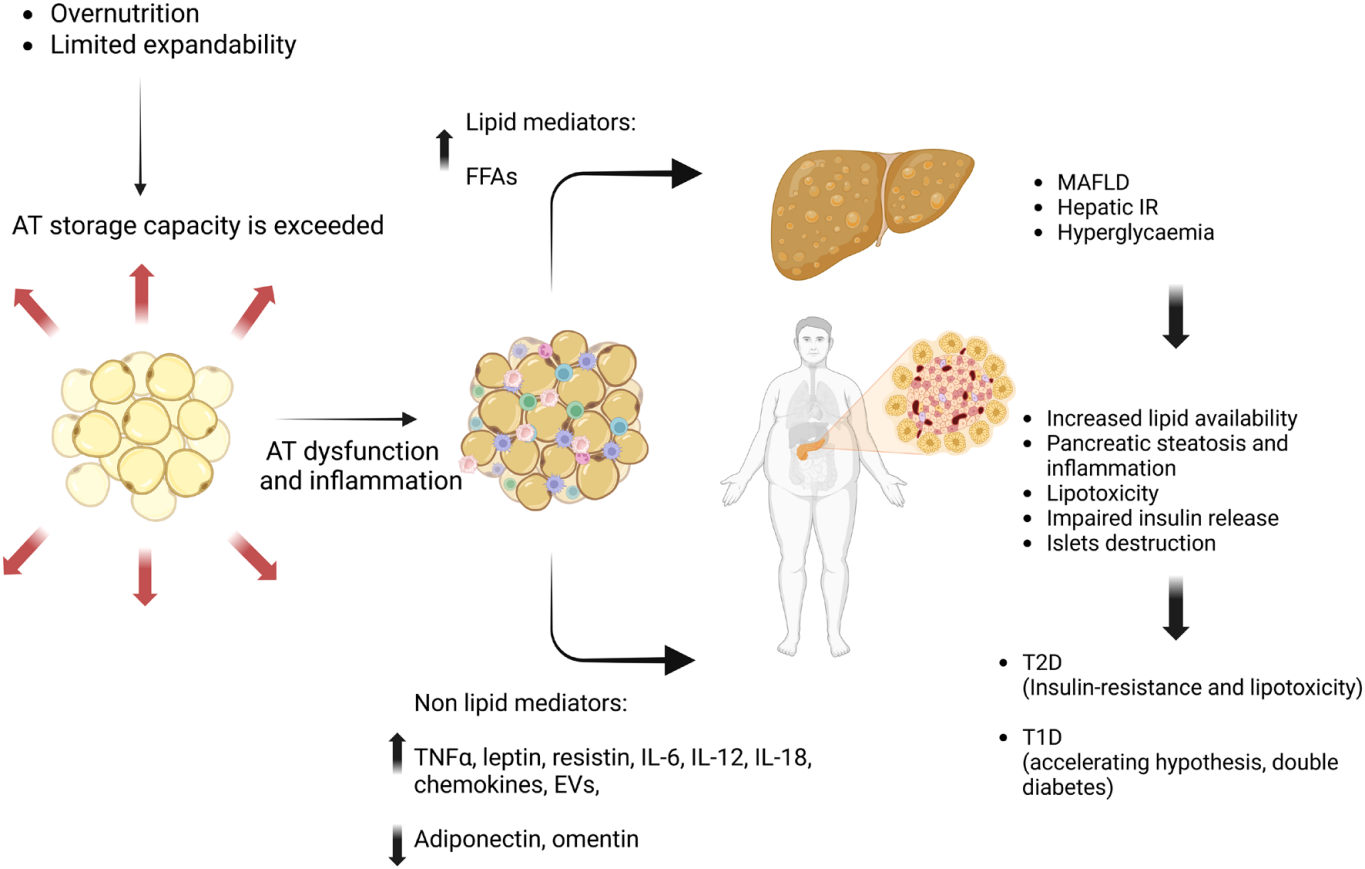
Diagram showing the adipocentric view of diabetes pathogenesis. In excessive energy supply, the expandability of adipose tissue (AT) is exceeded, and AT dysfunction and inflammation occur. The impaired AT sends lipid (free fatty acids) and non-lipid (adipokines, chemokines and EVs) detrimental mediators to the pancreas, leading to increased lipid availability, pancreatic lipid accumulation and inflammation, lipotoxicity, impaired insulin secretion and islet destruction. Moreover, it also leads to liver fat accumulation (MAFLD) and dysfunction, with subsequent hepatic IR and hyperglycaemia. Ultimately, this process evolves into systemic IR and type 2 diabetes pathogenesis. Moreover, it can also accelerate the appearance of T1D and aggravate its disease severity. AT, adipose tissue; EVs, extracellular vesicles; FFAs, free fatty acids; MAFLD, metabolic-associated fatty liver disease; T2D, type 2 diabetes mellitus; T1D, type 1 diabetes mellitus. Created with BioRender.com

**Figure 2:**
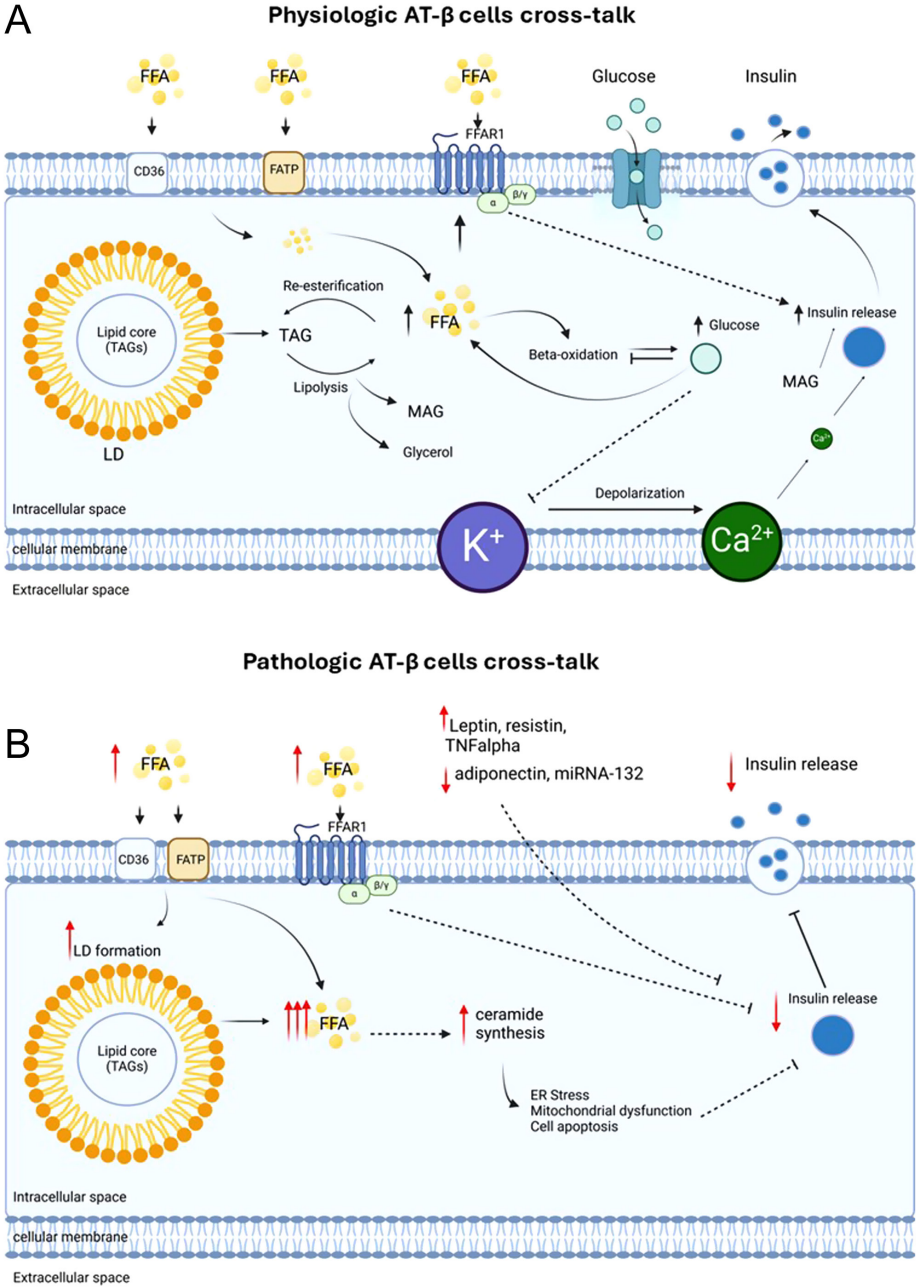
Physiologic (A) and pathologic (B) AT and β-cell crosstalk. (A) In physiologic conditions, a minimal level of FFAs is essential for normal GSIS. The increased intracellular glucose stimulates insulin secretion via closure of K+ channel, determining wave membrane depolarisation and intracellular Ca++ release which ultimately stimulate insulin granules release. At the extracellular level, FFAs stimulate GSIS by activating FFRA1, which increased insulin release. FFAs translocate in the cells facilitated by CD36 and FATP on the plasma membrane. The metabolism of intracellular lipid stimulates GSIS. The increased availability of FFAs contributes to activate FFAR1, β-oxidation and increase intracellular glucose levels, which stimulate insulin release. Moreover, FFAs accumulate in LDs in the form of TAGs. LDs degradation releases MAG and FFAs, which support GSIS as previously described. (B) In pathological conditions, AT dysfunction leads to increased availability of FFAs and non-lipid-mediators, such as leptin, resistin, TNF-α and to decreased levels of adiponectin and miRNA-132. The increased availability of FFAs determines lipotoxicity (ER stress, mitochondrial dysfunction and apoptosis) mainly by increasing *de novo* ceramides synthesis. All these signals together ultimately lead to β-cell dysfunction and insufficient insulin release. AT, adipose tissue; CD36, cluster of differentiation 36; FFAs, free fatty acids; FATP, fatty acid transport protein; FFRA1, free fatty acid receptor 1; GSIS, glucose-stimulated insulin secretion; LDs, lipid droplets; MAG, monoacylglycerol; TAGs, triacylglycerol. Created with BioRender.com

To sum up, AT and the pancreas communicate through an intricated complex of mediators, involving FFAs, lipid mediators, cytokines, EVs, adipokines and miRNAs (Figs. 1 and 2). In the case of AT dysfunction and abnormal distribution, all these signals together directly and indirectly contribute to pancreatic dysregulation and diabetes pathogenesis.

### Liver control of pancreatic function

In the attempt to connect AT dysfunction to diabetes pathogenesis and severity, the central role of liver control of lipid and glucose metabolism is worth mentioning. Under physiological conditions, the liver processes large quantities of FAs, but stores only small amounts in the form of TG, with steady-state TG contents of less than 5% (Browning *et al.* 2004, Targher *et al.* 2021). This is because the rates of acquisition of FA by uptake from the plasma and *de novo* synthesis within the liver are balanced by rates of FA oxidation and secretion into plasma as TG-enriched very low-density lipoprotein (VLDL-TAG). The relatively small quantities of TAG stored within the liver are localised in cytoplasmic LDs.

In the case of obesity, when AT expandability is reached, ectopic lipid flux is also increased towards the liver. The increased amount of FA directed to the liver exceeds its capacity to dispose of it, leading to hepatic fat accumulation. Metabolic-associated fatty liver disease (MAFLD) is the pathological hallmark of hepatic lipid deposition. MAFLD spectrum includes histologic features ranging from simple steatosis (MAFL) to steatohepatitis (metabolic-associated steatohepatitis (MASH)) and fibrosis, ultimately leading to cirrhosis (Azzu *et al.* 2020) (Fig. 1). The complex pathogenetic link from AT dysfunction to MAFLD has been extensively reviewed elsewhere (Azzu *et al.* 2020, Lee *et al.* 2023). In this paragraph, we will offer an overview of how the development of MAFLD is connected to diabetes pathogenesis. In fact, MAFLD and T2DM frequently coexist. A large meta-analysis of observational studies from 20 countries has estimated that the global prevalence of MAFLD in people with T2DM is ~56% (Morrison *et al.* 2019). Physiologically, in the fed state, insulin significantly suppresses hepatic glucose production by increasing net hepatic glycogen synthesis and suppressing hepatic gluconeogenesis (Prager *et al.* 1987, Cherrington *et al.* 1998); moreover, insulin is also a potent activator of DNL by activating SREBP-1c (Cherrington *et al.* 1998). When IR develops in the liver, there is a loss of suppression of hepatic glucose production, with subsequent hyperglycaemia and predisposition to diabetes. Despite the lipogenic activity of insulin, in the case of IR, DNL does not decrease but increases because of multiple inputs related to overnutrition able to drive the DNL programme (ChREBP, Mtorc1), leading to further hepatic fat accumulation and MAFLD. Several pathogenetic mechanisms are involved in this vicious circle, such as the increase in hepatic lipotoxic mediators, and in particular DAGs and ceramides, which directly and indirectly drive β-cell dysfunction and diabetes pathogenesis (Chavez & Summers 2012, Gadgil *et al.* 2022, Denimal *et al.* 2023).

### Improving AT function to restore pancreas function

Given the relationship between the AT and the pancreas, it is plausible that improving AT function is a valuable option to protect β cells, prevent diabetes development and maintain glucose homeostasis. Moreover, international guidelines for the treatment of diabetes promote obesity management in patients with diabetes and obesity (American Diabetes Association 2018), with lifestyle dietary interventions and metabolic bariatric surgery being able to prevent, control and even reverse diabetes (Diabetes Prevention Program Research Group 2002, Lindström *et al.* 2006, Diabetes Prevention Program Research Group 2009, Hopper *et al.* 2011). In particular, data from the Diabetes Remission Clinical Trial (DIRECT) highlighted that diet-induced weight loss of over 10 kg in people with obesity and T2D was able to decrease liver and pancreatic fat content, as well as plasma TGs. However, subjects who returned to non-diabetic glucose control (responders) had shorter diabetes duration, suggesting that diabetes can be reverted as long as β-cell function is preserved (Taylor *et al.* 2018).

The growing market for oral antidiabetic medications has brought new pharmaceutical strategies targeting obesity and hyperglycaemia based on glucagon-like polypeptide 1 (GLP1) agonists. These molecules, such as liraglutide and semaglutide, are effective for diabetes management and cardiovascular complications. Their effectiveness is reflected by their capacity to reduce body weight, AT inflammation and improve body fat distribution (Wang *et al.* 2023). Moreover, GLP1 agonists have been proven to reduce postprandial lipaemia (Hermansen *et al.* 2013, Voukali *et al.* 2014). Pointing in this direction, the newly available antidiabetic medication, Tirzepatide, a glucose-dependent insulinotropic polypeptide (GIP)/GLP-1 dual agonist, shows even more valuable effects on glycaemic control and body weight management (Jastreboff *et al.* 2022). Interestingly, GIP receptors are highly expressed in the AT, where they stimulate fatty acid oxidation and enhance insulin sensitivity (Yip & Wolfe 1999), suggesting that the AT might mediate – at least partially – the effectiveness of this molecule. The SURMOUNT-2 study showed that 72 weeks of treatment with once-weekly Tirzepatide 10 mg or 15 mg resulted in a significant mean weight loss versus placebo of 9.6% and 11.6%, respectively, and a mean reduction of HbA1c from 8% to 5.9%, in 1514 adults with obesity and T2D (Garvey *et al.* 2016). Moreover, a recent phase 1 study on 117 participants showed that tirzepatide treatment compared to semaglutide was able to improve more significantly β cell function, evaluated as amelioration of clamp-derived total insulin secretion rate and insulin sensitivity (Heise *et al.* 2022). These data highlight the significant efficacy of therapies targeting obesity and diabetes and the need to develop new targets and alternative drugs for both conditions.

### Improving AT function using thiazolidinediones

Among the antidiabetic medications, synthetic peroxisome proliferator-activated receptor γ (PPARγ) agonists (e.g. thiazolidinediones, TZDs or glitazones) represent the most considerable example of how improving AT function is beneficial for diabetes management. PPARγ, particularly the PPARγ2 isoform, is a nuclear hormone receptor superfamily member highly expressed in the AT, where it is considered the master regulator of adipogenesis and promotes insulin sensitivity, lipogenesis, lipid storage and glucose metabolism (Semple *et al.* 2006). Preclinical studies showed that PPARγ2 is needed to promote healthy AT expansion, promoting redistribution of fat mass towards the scWAT, in mice but is also required for the β-cell expansion adaptive response to IR (Medina-Gomez *et al.* 2007) In line with the critical role of PPARγ in preventing metabolic disorders, epidemiological studies showed that specific polymorphism of the PPARγ gene affect metabolic traits and susceptibility to diabetes (Heikkinen *et al.* 2007). Moreover, a set of mutations in the PPARγ gene is associated with familial partial lipodystrophies, characterised by a deficiency of limb and gluteal fat in favour of abdominal and visceral fat supporting its role in the regulation of AT distribution (Garg 2004). Pointing in the same direction, TZDs are currently prescribed worldwide to treat hyperglycaemia and diabetes. The antidiabetic effect of TZDs is traditionally explained by the PPARγ-mediated expansion of subcutaneous AT, resulting in a reduction in systemic lipid concentrations and the subsequent reversal of ‘lipotoxicity’ in non-adipose tissues, such as pancreatic β cells (Bays *et al.* 2004). Recent estimates of adipogenesis in obese individuals treated with pioglitazone (White *et al.* 2021) confirm earlier morphological observations (McLaughlin *et al.* 2010) that TZDs drive AT expansion by stimulating the adipocyte hyperplasia. As a result of this mode of action, TZDs promote healthy fat mass gain, improving dyslipidaemia, insulin sensitivity and glycaemic control (Kelly *et al.* 1999, Mori *et al.* 1999, Miyazaki *et al.* 2001, Shadid & Jensen 2003), confirming the role of AT expandability in metabolic impairment pathogenesis. Therefore, at least partially, the beneficial action of TZDs can be explained by their increased AT mass and insulin sensitivity, leading to a reduction in AT lipolysis and subsequent lowering of circulating FFA and triglycerides. As a result, the lipid bioavailability for the pancreas is limited, which should reduce pancreatic steatosis.

However, the use of TZDs in clinics has drastically reduced since 2009. Accordingly, rosiglitazone has been removed from the market in several countries (Cohen 2010) following the results of the RECORD trial 2019, showing an increased risk of heart failure and fractures in women (Home *et al.* 2009). A meta-analysis in 2017 confirmed the suspicion that rosiglitazone use was associated with an increased risk of myocardial infarction and cardiovascular death (Nissen & Wolski 2007). The Food and Drug Administration restricted rosiglitazone sales between 2011 and 2013 with a risk evaluation and medications strategy and then allowed its sale again afterwards (U.S. Food and Drug Administration 2015). No such restriction has ever been placed on pioglitazone, leading to glycaemic control amelioration and improvements in HbA1c, insulin sensitivity and lipid profile (Herz *et al.* 2003). As a proof of its particular effectiveness in IR management, evidence supports that pioglitazone was associated with a more significant improvement of insulin sensitivity than DPP4 inhibitors (Bae *et al.* 2019), and a significant improvement of β-cell function assessed by euglycaemic insulin clamp (Gastaldelli *et al.* 2007). However, in the clinical setting, pioglitazone is limited by its adverse effects on heart failure, peripheral oedema and bone fracture risk (Liao *et al.* 2017). In contrast, it is associated with a reduced risk of non-fatal myocardial infarction, stroke, and cardiovascular death (MACE) (Liao *et al.* 2017). Therefore, there is a need to develop new treatments to improve AT function without the cardiovascular side effects associated with TZDs.

Altogether, these data suggest that the AT represents a significant target for diabetes prevention and treatment, mediating, at least in part, the effects of novel and effective antidiabetic medications. This further proves the importance of AT health and expandability in lipotoxicity and β-cell impairment.

### Adipose tissue dysfunction is also significant in autoimmune diabetes

Although T1D is traditionally considered a disease of lean people, overweight and obesity are becoming increasingly more common in individuals with T1D, defining a new phenotype of patients with T1D, often referred to as ‘double diabetes’ which is characterised by a mixture of T1D and T2D (Khawandanah 2019). The prevalence of obesity among patients with T1D has been reported to be between 2.8% and 37.1% (Merger *et al.* 2016, Mishra *et al.* 2018), representing an emerging clinical issue about which our knowledge is still poor.

Whereas T1D is caused by autoimmune β-cell destruction, which leads to absolute insulin deficiency, with insulin treatment classically needed from the onset of the disease, AT dysfunction might influence and amplify the pathogenesis and severity of T1D. It has been suggested that there is a likely association between higher birthweight, childhood obesity and higher BMI with an increased risk of childhood-onset T1D (Betts *et al.* 2005, Verbeeten *et al.* 2011, Wilkin 2009). A retrospective study on 168 people presenting with T1D showed that both pre-onset and post-diagnosis BMI were inversely correlated with age at onset (Betts *et al.* 2005), and a meta-analysis of four studies confirmed the association between obesity and development of T1D (Verbeeten *et al.* 2011). One hypothesis to explain this paradoxical finding is the ‘accelerator hypothesis’, according to which obesity and lipotoxicity impose more stress on β-cells, making them more vulnerable to earlier and stronger autoimmune destruction (Wilkin 2009).

Beyond being implicated in the earlier appearance of the disease, AT dysfunction is a risk factor for T1D severity. Patients with double diabetes are characterised by the presence of higher IR and requirements, greater cardiometabolic risk and an enhanced risk of developing chronic complications, such as dyslipidaemia, hypertension, atherosclerosis and heart failure (Kietsiriroje *et al.* 2019, Van der Schueren *et al.* 2021). Moreover, obesity represents a risk factor for worse glycaemic control in T1D (Wing & Cleary 1988).

Even though it is reasonable that targeting AT might benefit T1D, no specific guidelines are available for treating obesity and T1D. Lifestyle modifications classically recommended in patients with obesity represent a significant challenge in the clinical routine management of patients with T1D, given the difficulties in managing hypocaloric diets and physical activity in insulin-dependent patients. Moreover, data evaluating drugs targeting AT in patients with T1D are still limited but suggest that these patients may benefit from pharmaceutical interventions targeting AT. For example, the REMOVAL study showed that the use of metformin as an adjunct therapy in T1D led to modest weight loss (Petrie *et al.* 2017); the ADJUNT studies reported that liraglutide could lead to a dose-dependent weight loss in people with T1D, however, accompanied by a slight increase in severe hypoglycaemia (Ghanim *et al.* 2020, Mathieu *et al.* 2016); noteworthy, a recent small case series showed that semaglutide treatment in early T1D was able to eliminate prandial insulin administration need and basal insulin in most patients, suggesting its potential role in delaying pancreatic β cells destruction (Dandona *et al.* 2023). Finally, pramlintide, a synthetic analogue of human amylin, is the only FDA-approved therapy for T1D (Childs 2006), and it has been shown to reduce HbA1c, daily insulin doses, and postprandial glucose concentrations, with a concomitant modest effect on body weight (Ratner *et al.* 2004).

Given the increasing overlap between obesity and T1D, scientific attention should be devoted to understanding the relationship between AT dysfunction and T1D pathogenesis and severity to offer a more personalised and targeted approach to this particular category of frail patients.

## Conclusion

Obesity and diabetes represent two increasing and invalidating chronic diseases that often coexist. The increasing prevalence of obesity worldwide is closely associated with a similar dramatic trend in the incidence of diabetes (GBD 2021 Diabetes Collaborators 2023), and obesity is widely recognised as a predisposing factor for T2D (Klein *et al.* 2022).

In this review, we approach the development of β-cell dysfunction and diabetes from an adipocentric point of view, as summarised in Fig. 1. Considering the well-described deleterious effect of chronic exposure to high levels of FA on β-cell dysfunction, we believe that limiting lipid supply to the pancreas by improving AT function offers a valuable and effective target to prevent, control and reverse diabetes itself, being relevant not only in type 2 but also in type 1 diabetes mellitus.

## Declaration of interest

The authors declare that there is no conflict of interest that could be perceived as prejudicing the impartiality of the study reported.

## Funding

This work was supported by the Addenbrookes Charitable Trust Clinical Research Fellowship (grant number G113726) and by the British Heart Foundationhttp://dx.doi.org/10.13039/501100000274 (RG/F/23/110110).

## References

[bib2] Ahima RS & Lazar MA 2008 Adipokines and the peripheral and neural control of energy balance. Molecular Endocrinology 22 1023–1031. (10.1210/me.2007-0529) 18202144 PMC2366188

[bib3] Ahrèn B & Larsson H 1997 Leptin: a regulator of islet function?: its plasma levels correlate with glucagon and insulin secretion in healthy women. Metabolism: Clinical and Experimental 46 1477–1481. (10.1016/s0026-0495(9790152-9) 9439547

[bib239] Al-Mrabeh A Zhyzhneuskaya SV Peters C Barnes AC Melhem S Jesuthasan A Aribisala B Hollingsworth KG Lietz G Mathers JCet al. 2020 Hepatic lipoprotein export and remission of human type 2 diabetes after weight loss. Cell Metabolism 31 233–249.e4. (10.1016/j.cmet.2019.11.018) 31866441

[bib4] Al-Salam S Rashed H & Adeghate E 2011 Diabetes mellitus is associated with an increased expression of resistin in human pancreatic islet cells. Islets 3 246–249. (10.4161/isl.3.5.16427) 21750415

[bib5] American Diabetes Association (ADA) 2018 Obesity management for the treatment of type 2 diabetes: standards of medical care in diabetes-2018. Diabetes Care 41(Suppl 1) S65–S72. (10.2337/dc18-s007) 29222378

[bib6] Armour SL Frueh A Chibalina MV Dou H Argemi-Muntadas L Hamilton A Katzilieris-Petras G Carmeliet P Davies B Moritz T, *et al.* 2023 Glucose controls glucagon secretion by regulating fatty acid oxidation in pancreatic α-cells. Diabetes 72 1446–1459. (10.2337/db23-0056) 37494670 PMC10545563

[bib8] Arner P & Rydén M 2015 Fatty acids, obesity and insulin resistance. Obesity Facts 8 147–155. (10.1159/000381224) 25895754 PMC5644864

[bib7] Arner P Hellström L Wahrenberg H & Brönnegård M 1990 Beta-adrenoceptor expression in human fat cells from different regions. Journal of Clinical Investigation 86 1595–1600. (10.1172/JCI114880) 2173724 PMC296908

[bib9] Attané C Peyot ML Lussier R Poursharifi P Zhao S Zhang D Morin J Pineda M Wang S Dumortier O, *et al.* 2016 A beta cell ATGL-lipolysis/adipose tissue axis controls energy homeostasis and body weight via insulin secretion in mice. Diabetologia 59 2654–2663. (10.1007/s00125-016-4105-2) 27677764 PMC6518076

[bib10] Azzu V Vacca M Virtue S Allison M & Vidal-Puig A 2020 Adipose tissue-liver cross talk in the control of whole-body metabolism: implications in nonalcoholic fatty liver disease. Gastroenterology 158 1899–1912. (10.1053/j.gastro.2019.12.054) 32061598

[bib11] Bae J Kim G Lee YH Lee BW Kang ES & Cha BS 2019 Differential effects of thiazolidinediones and dipeptidyl peptidase-4 inhibitors on insulin resistance and β-cell function in type 2 diabetes mellitus: a propensity score-matched analysis. Diabetes Therapy 10 149–158. (10.1007/s13300-018-0541-y) 30506494 PMC6349276

[bib12] Baldwin AC Green CD Karl Olson LK Moxley MA & Corbett JA 2012 A role for aberrant protein palmitoylation in FFA-induced ER stress and β-cell death. American Journal of Physiology. Endocrinology and Metabolism 302 E1390–E1398. (10.1152/ajpendo.00519.2011) 22436701 PMC3378068

[bib13] Barnes AS 2011 The epidemic of obesity and diabetes: trends and treatments. Texas Heart Institute Journal 38 142–144. 21494521 PMC3066828

[bib14] Bays H Mandarino L & DeFronzo RA 2004 Role of the adipocyte, free fatty acids, and ectopic fat in pathogenesis of type 2 diabetes mellitus: peroxisomal proliferator-activated receptor agonists provide a rational therapeutic approach. Journal of Clinical Endocrinology and Metabolism 89 463–478. (10.1210/jc.2003-030723) 14764748

[bib15] Becattini B Molinaro A Henricsson M Boren J & Solinas G 2023 Adipocyte PI3K links adipostasis with basal insulin secretion through an adipoincretin effect. bioRxiv [epub]. (10.1101/2023.04.24.538076) 38656871

[bib16] Betts P Mulligan J Ward P Smith B & Wilkin T 2005 Increasing body weight predicts the earlier onset of insulin-dependant diabetes in childhood: testing the ‘accelerator hypothesis’. Diabetic Medicine 22 144–151. (10.1111/j.1464-5491.2004.01368.x) 15660730

[bib17] Binnert C Pachiaudi C Beylot M Croset M Cohen R Riou JP & Laville M 1996 Metabolic fate of an oral long-chain triglyceride load in humans. American Journal of Physiology 270 E445–E450. (10.1152/ajpendo.1996.270.3.E445) 8638691

[bib18] Boden G 2008 Obesity and free fatty acids. Endocrinology and Metabolism Clinics of North America 37 635–646. (10.1016/j.ecl.2008.06.007) 18775356 PMC2596919

[bib19] Bollheimer LC Landauer HC Troll S Schweimer J Wrede CE Scholmerich J & Buettner R 2004 Stimulatory short-term effects of free fatty acids on glucagon secretion at low to normal glucose concentrations. Metabolism: Clinical and Experimental 53 1443–1448. (10.1016/j.metabol.2004.06.011) 15536599

[bib20] Boslem E McIntosh G Preston AM Bartely C Busch AK Fuller M Laybutt DR Meikle PJ & Biden TJ 2011 A lipidomic screen of palmitate-treated MIN6 β-cells links sphingolipid metabolites with endoplasmic reticulum (ER) stress and impaired protein trafficking. Biochemical Journal 435 267–276. (10.1042/BJ20101867) 21265737

[bib21] Boslem E Weir JM MacIntosh G Sue N Cantley J Meikle PJ & Biden TJ 2013 Alteration of endoplasmic reticulum lipid rafts contributes to lipotoxicity in pancreatic β-cells. Journal of Biological Chemistry 288 26569–26582. (10.1074/jbc.M113.489310) 23897822 PMC3772204

[bib22] Briaud I Kelpe CL Johnson LM Tran POT & Poitout V 2002 Differential effects of hyperlipidemia on insulin secretion in islets of langerhans from hyperglycemic versus normoglycemic rats. Diabetes 51 662–668. (10.2337/diabetes.51.3.662) 11872664

[bib23] Brown JEP Onyango DJ & Dunmore SJ 2007 Resistin down-regulates insulin receptor expression, and modulates cell viability in rodent pancreatic beta-cells. FEBS Letters 581 3273–3276. (10.1016/j.febslet.2007.06.031) 17597619

[bib24] Browning JD Szczepaniak LS Dobbins R Nuremberg P Horton JD Cohen JC Grundy SM & Hobs HH 2004 Prevalence of hepatic steatosis in an urban population in the United States: impact of ethnicity. Hepatology 40 1387–1395. (10.1002/hep.20466) 15565570

[bib25] Bush NC Triay JM Gathaiya NW Hames KC & Jensen MD 2014 Contribution of very low-density lipoprotein triglyceride fatty acids to postabsorptive free fatty acid flux in obese humans. Metabolism: Clinical and Experimental 63 137–140. (10.1016/j.metabol.2013.09.008) 24140092 PMC3859725

[bib26] Cantley J Davenport A Vetterli L Nemes NJ Whitworth PT Boslem E Thai LM Mellett N Meikle PJ Hoenh KL, *et al.* 2019 Disruption of beta cell acetyl-CoA carboxylase-1 in mice impairs insulin secretion and beta cell mass. Diabetologia 62 99–111. (10.1007/s00125-018-4743-7) 30334081 PMC6290731

[bib27] Cantley NW Lonnen K Kyrou I Tahrani AA & Kahal H 2021 The association between overweight/obesity and double diabetes in adults with type 1 diabetes; a cross-sectional study. BMC Endocrine Disorders 21 187. (10.1186/s12902-021-00851-1) 34530819 PMC8447500

[bib28] Carpentier AC 2008 Postprandial fatty acid metabolism in the development of lipotoxicity and type 2 diabetes. Diabetes and Metabolism 34 97–107. (10.1016/j.diabet.2007.10.009) 18353699

[bib29] Cen J Sargsyan E & Bergsten P 2016 Fatty acids stimulate insulin secretion from human pancreatic islets at fasting glucose concentrations via mitochondria-dependent and -independent mechanisms. Nutrition and Metabolism 13 59. (10.1186/s12986-016-0119-5) 27582778 PMC5006523

[bib31] Chavez JA & Summers SA 2003 Characterizing the effects of saturated fatty acids on insulin signaling and ceramide and diacylglycerol accumulation in 3T3-L1 adipocytes and C2C12 myotubes. Archives of Biochemistry and Biophysics 419 101–109. (10.1016/j.abb.2003.08.020) 14592453

[bib32] Chavez JA & Summers SA 2012 A ceramide-centric view of insulin resistance. Cell Metabolism 15 585–594. (10.1016/j.cmet.2012.04.002) 22560211

[bib30] Chavez JA Knotts TA Wang LP Li G Dobrowsky RT Florant GL & Summers SA 2003 AA role for ceramide, but not diacylglycerol, in the antagonism of insulin signal transduction by saturated fatty acids. Journal of Biological Chemistry 278 10297–10303. (10.1074/jbc.M212307200) 12525490

[bib33] Cherrington AD Edgerton D & Sindelar DK 1998 The direct and indirect effects of insulin on hepatic glucose production in vivo. Diabetologia 41 987–996. (10.1007/s001250051021) 9754815

[bib34] Childs BP 2006 Pramlintide use in type 1 diabetes resulting in less hypoglycemia. Diabetes Spectrum 19 50–52. (10.2337/diaspect.19.1.50)

[bib35] Chin SO Hwang YC Cho IJ Jeong IK Ahn KJ & Chung HY 2021 Pancreatic fat accumulation is associated with decreased β-cell function and deterioration in glucose tolerance in Korean adults. Diabetes/Metabolism Research and Reviews 37 e3425. (10.1002/dmrr.3425) 33258260

[bib36] Christensen AA & Gannon M 2019 The beta cell in type 2 diabetes. Current Diabetes Reports 19 81. (10.1007/s11892-019-1196-4) 31399863

[bib37] Cnop M Grupping A Hoorens A Bouwens L Pipeleers-Marichal M & Pipeleers D 2000 Endocytosis of low-density lipoprotein by human pancreatic β cells and uptake in lipid-storing vesicles, which increase with age. American Journal of Pathology 156 237–244. (10.1016/s0002-9440(1064724-4) 10623672 PMC1868647

[bib38] Cnop M Havel PJ Utzschneider KM Carr DB Sinha MK Boyko EJ Retzlaff BM Knopp RH Brunzell JD & Kahn SE 2003 Relationship of adiponectin to body fat distribution, insulin sensitivity and plasma lipoproteins: evidence for independent roles of age and sex. Diabetologia 46 459–469. (10.1007/s00125-003-1074-z) 12687327

[bib40] Cnop M Welsh N Jonas JC Jörns A Lenzen S & Eizirik DL 2005 Mechanisms of pancreatic beta-cell death in type 1 and type 2 diabetes: many differences, few similarities. Diabetes 54(Supplement 2) S97–107. (10.2337/diabetes.54.suppl_2.s97) 16306347

[bib39] Cnop M Ladriere L Hekerman P Ortis F Cardozo AK Dogusan Z Flamez D Boyce M Yuan J & Eizirik DL 2007 Selective inhibition of eukaryotic translation initiation factor 2 alpha dephosphorylation potentiates fatty acid-induced endoplasmic reticulum stress and causes pancreatic beta-cell dysfunction and apoptosis. Journal of Biological Chemistry 282 3989–3997. (10.1074/jbc.M607627200) 17158450

[bib41] Cohen D 2010 Rosiglitazone: what went wrong. BMJ 341 c4848–c4848. (10.1136/bmj.c4848) 20819889

[bib42] Colditz GA Willett WC Rotnitzky A & Manson JE 1995 Weight gain as a risk factor for clinical diabetes mellitus in women. Annals of Internal Medicine 122 481–486. (10.7326/0003-4819-122-7-199504010-00001) 7872581

[bib43] Cui X You L Zhu L Wang X Zhou Y Li Y Wen J Xia Y Wang X Ji C, *et al.* 2018 Change in circulating microRNA profile of obese children indicates future risk of adult diabetes. Metabolism: Clinical and Experimental 78 95–105. (10.1016/j.metabol.2017.09.006) 28966078

[bib44] Cunha DA Hekerman P Ladrière L Bazarra-Castro A Ortis F Wakeham MC Moore F Rasschaert J Cardozo AK Bellomo E, *et al.* 2008 Initiation and execution of lipotoxic ER stress in pancreatic β-cells. Journal of Cell Science 121 2308–2318. (10.1242/jcs.026062) 18559892 PMC3675788

[bib45] Dai C Kayton NS Shostak A Poffenberger G Cyphert HA Aramandla R Thompson C Papagiannis IG Emfinger C Shiota M, *et al.* 2016 Stress-impaired transcription factor expression and insulin secretion in transplanted human islets. Journal of Clinical Investigation 126 1857–1870. (10.1172/JCI83657) 27064285 PMC4855919

[bib46] Dandona P Chaudhuri A & Ghanim H 2023 Semaglutide in early type 1 diabetes. New England Journal of Medicine 389 958–959. (10.1056/NEJMc2302677) 37672701

[bib47] Davoren PM Kelly W Gries FA Hubinger A Whately-Smith C & Alberti KGMM 1998 Long-term effects of a sustained-release preparation of acipimox on dyslipidemia and glucose metabolism in non-insulin-dependent diabetes mellitus. Metabolism: Clinical and Experimental 47 250–256. (10.1016/s0026-0495(9890252-9) 9500558

[bib48] De Boer IH Sibley SD Kestenbaum B Sampson JN Young B Cleary PA Steffes MW Weiss NS Brunzell JD & Diabetes Control and Complications Trial/Epidemiology of Diabetes Interventions and Complications Study Research Group 2007 Central obesity, incident microalbuminuria, and change in creatinine clearance in the epidemiology of diabetes interventions and complications study. Journal of the American Society of Nephrology 18 235–243. (10.1681/ASN.2006040394) 17151331 PMC2622719

[bib49] Denimal D Béland-Bonenfant S Pais-de-Barros JP Rouland A Bouillet B Duvillad L Verges B & Petit JM 2023 Plasma ceramides are associated with MRI-based liver fat content but not with noninvasive scores of liver fibrosis in patients with type 2 diabetes. Cardiovascular Diabetology 22 310. (10.1186/s12933-023-02049-2) 37940926 PMC10634084

[bib50] Diabetes Prevention Program Research Group, Knowler WC Barrett-Connor E Fowler SE Hamman RF Lachin JM Walker EA & Nathan DM 2002 Reduction in the incidence of type 2 diabetes with lifestyle intervention or metformin. New England Journal of Medicine 346 393–403. (10.1056/NEJMoa012512) 11832527 PMC1370926

[bib51] Diabetes Prevention Program Research Group, Knowler WC Fowler SE Hamman RF Christophi CA Hoffman HJ Brenneman AT Brown-Friday JO Goldberg R Venditti E, *et al.* 2009 10-year follow-up of diabetes incidence and weight loss in the Diabetes Prevention Program Outcomes Study. Lancet 374 1677–1686. (10.1016/S0140-6736(0961457-4) 19878986 PMC3135022

[bib52] Drolet R Bélanger C Fortier M Huot C Mailloux J Legarè D & Tchernof A 2009 Fat depot-specific impact of visceral obesity on adipocyte adiponectin release in women. Obesity (Silver Spring) 17 424–430. (10.1038/oby.2008.555) 19219061

[bib53] Duncan RE Ahmadian M Jaworski K Sarkadi-Nagy E & Sul HS 2007 Regulation of lipolysis in adipocytes. Annual Review of Nutrition 27 79–101. (10.1146/annurev.nutr.27.061406.093734) PMC2885771 17313320

[bib55] Eizirik DL Cardozo AK & Cnop M 2008 The role for endoplasmic reticulum stress in diabetes mellitus. Endocrine Reviews 29 42–61. (10.1210/er.2007-0015) 18048764

[bib56] Elouil H Bensellam M Guiot Y Vander Mierde D Pascal SMA Schuit FC & Jonas JC 2007 Acute nutrient regulation of the unfolded protein response and integrated stress response in cultured rat pancreatic islets. Diabetologia 50 1442–1452. (10.1007/s00125-007-0674-4) 17497122

[bib57] Fielding BA Callow J Owen RM Samra JS Matthews DR & Frayn KN 1996 Postprandial lipemia: the origin of an early peak studied by specific dietary fatty acid intake during sequential meals. American Journal of Clinical Nutrition 63 36–41. (10.1093/ajcn/63.1.36) 8604667

[bib237] Flodgren E Olde B Meidute-Abaraviciene S Winzell MS Ahrén B & Salehi A 2007 GPR40 is expressed in glucagon producing cells and affects glucagon secretion. Biochemical and Biophysical Research Communications 354 240–245. (10.1016/j.bbrc.2006.12.193) 17214971

[bib58] Fontana L Eagon JC Trujillo ME Scherer PE & Klein S 2007 Visceral fat adipokine secretion is associated with systemic inflammation in obese humans. Diabetes 56 1010–1013. (10.2337/db06-1656) 17287468

[bib59] Frayn KN 2000 Visceral fat and insulin resistance--causative or correlative? British Journal of Nutrition 83(Supplement 1) S71–S77. (10.1017/s0007114500000982) 10889795

[bib60] Frayn KN 2002 Adipose tissue as a buffer for daily lipid flux. Diabetologia 45 1201–1210. (10.1007/s00125-002-0873-y) 12242452

[bib61] Fraze E Donner CC Swislocki ALM Chiou YAM Chen YDI & Reaven GM 1985 Ambient plasma free fatty acid concentrations in noninsulin-dependent diabetes mellitus: evidence for insulin resistance. Journal of Clinical Endocrinology and Metabolism 61 807–811. (10.1210/jcem-61-5-807) 3900120

[bib63] Fryk E, Olausson J Mossberg K Strindberg L Schmelz M Brogren H Gan LM Piazza S Provenzani A Becattini B, *et al.* 2021 Hyperinsulinemia and insulin resistance in the obese may develop as part of a homeostatic response to elevated free fatty acids: a mechanistic case-control and a population-based cohort study. EBiomedicine 65 103264. (10.1016/j.ebiom.2021.103264) 33712379 PMC7992078

[bib64] Gadgil MD Sarkar M Sands C Lewis MR Herrington DM & Kanaya AM 2022 Associations of NAFLD with circulating ceramides and impaired glycemia. Diabetes Research and Clinical Practice 186 109829. (10.1016/j.diabres.2022.109829) 35292328 PMC9082931

[bib65] García-Ruiz C Colell A Morales A Calvo M Enrich C & Fernández-Checa JC 2002 Trafficking of ganglioside GD3 to mitochondria by tumor necrosis factor-α. Journal of Biological Chemistry 277 36443–36448. (10.1074/jbc.M206021200) 12118012

[bib66] Garg A 2004 Acquired and inherited lipodystrophies. New England Journal of Medicine 350 1220–1234. (10.1056/NEJMra025261) 15028826

[bib67] Garg A 2011 Lipodystrophies: genetic and acquired body fat disorders. Journal of Clinical Endocrinology and Metabolism 96 3313–3325. (10.1210/jc.2011-1159) 21865368 PMC7673254

[bib245] Garvey WT Frias JP Jastreboff AM le Roux CW Sattar N Aizenberg D Mao H Zhang S Ahmad NN Bunck MCet al. 2023 Tirzepatide once weekly for the treatment of obesity in people with type 2 diabetes (SURMOUNT-2): a double-blind, randomised, multicentre, placebo-controlled, phase 3 trial. Lancet 402 613–626. (10.1016/s0140-6736(2301200-x 37385275

[bib68] Gastaldelli A Ferrannini E Miyazaki Y Matsuda M Mari A & DeFronzo RA 2007 Thiazolidinediones improve beta-cell function in type 2 diabetic patients. American Journal of Physiology. Endocrinology and Metabolism 292 E871–E883. (10.1152/ajpendo.00551.2006) 17106061

[bib69] Gavrilova O Marcus-Samuels B Graham D Kim JK Shulman GI Castle AL Vinson C Eckhaus M & Reitman ML 2000 Surgical implantation of adipose tissue reverses diabetes in lipoatrophic mice. Journal of Clinical Investigation 105 271–278. (10.1172/JCI7901) 10675352 PMC377444

[bib70] GBD 2021 Diabetes Collaborators 2023 Global, regional, and national burden of diabetes from 1990 to 2021, with projections of prevalence to 2050: a systematic analysis for the Global Burden of Disease Study 2021. Lancet 402 203–234. (10.1016/S0140-6736(2301301-6) 37356446 PMC10364581

[bib71] Gealekman O Guseva N Hartigan C Apotheker S Gorgoglione M Gurav K Tran KV Straubhaar J Nicoloro S Czech MP, *et al.* 2011 Depot-specific differences and insufficient subcutaneous adipose tissue angiogenesis in human obesity. Circulation 123 186–194. (10.1161/CIRCULATIONAHA.110.970145). [Correction: Circulation 2019 140 e693. (10.1161/CIRCULATIONAHA.110.970145)] 21200001 PMC3334340

[bib72] Gesmundo I Pardini B Gargantini E Gamba G Birolo G Fanciulli A Banfi D Congiusta N Favaro E Deregibus MC, *et al.* 2021 Adipocyte-derived extracellular vesicles regulate survival and function of pancreatic β cells. JCI Insight 6 1–17. (10.1172/jci.insight.141962) PMC8021102 33539327

[bib73] Ghanim H Batra M Green K Abuaysheh S Hejna J Makdissi A Borowski R Kuhadiva ND Chaudhuri A & Dandona P 2020 Liraglutide treatment in overweight and obese patients with type 1 diabetes: a 26-week randomized controlled trial; mechanisms of weight loss. Diabetes, Obesity and Metabolism 22 1742–1752. (10.1111/dom.14090) 32424935

[bib74] Ginsberg HN Packard CJ Chapman MJ Borén J Aguilar-Salinas CA Averna M Ference BA Gaudet D Hegele RA Kersten S, *et al.* 2021 Triglyceride-rich lipoproteins and their remnants: metabolic insights, role in atherosclerotic cardiovascular disease, and emerging therapeutic strategies-a consensus statement from the European Atherosclerosis Society. European Heart Journal 42 4791–4806. (10.1093/eurheartj/ehab551) 34472586 PMC8670783

[bib75] Gjedsted J Gormsen LC Nielsen S Schmitz O Djurhuus CB Keiding S Ørskov H Tønnesen E & Møller N 2007 Effects of a 3-day fast on regional lipid and glucose metabolism in human skeletal muscle and adipose tissue. Acta Physiologica 191 205–216. (10.1111/j.1748-1716.2007.01740.x) 17784905

[bib76] Gluchowski NL Becuwe M Walther TC & Farese Jr RV 2017 Lipid droplets and liver disease: from basic biology to clinical implications. Nature Reviews. Gastroenterology and Hepatology 14 343–355. (10.1038/nrgastro.2017.32) 28428634 PMC6319657

[bib77] Grujic D Susulic VS Harper ME Himms-Hagen J Cunningham BA Corkey BE & Lowell BB 1997 β3-adrenergic receptors on white and brown adipocytes mediate β3- selective agonist-induced effects on energy expenditure, insulin secretion, and food intake: a study using transgenic and gene knockout mice. Journal of Biological Chemistry 272 17686–17693. (10.1074/jbc.272.28.17686) 9211919

[bib78] Hamilton JA & Kamp F 1999 How are free fatty acids transported in membranes? Is it by proteins or by free diffusion through the lipids? Diabetes 48 2255–2269. (10.2337/diabetes.48.12.2255) 10580412

[bib79] Heikkinen S Auwerx J & Argmann CAA 2007 PPARgamma in human and mouse physiology. Biochimica et Biophysica Acta 1771 999–1013. (10.1016/j.bbalip.2007.03.006) 17475546 PMC2020525

[bib80] Heilbronn LK Rood J Janderova L Albu JB Kelley DE Ravussin E & Smith SR 2004 Relationship between serum resistin concentrations and insulin resistance in nonobese, obese, and obese diabetic subjects. Journal of Clinical Endocrinology and Metabolism 89 1844–1848. (10.1210/jc.2003-031410) 15070954

[bib81] Heise T Mari A DeVries JH Urva S Li J Pratt EJ Coskun T Thomas MK Mather KJ Haupt A, *et al.* 2022 Effects of subcutaneous tirzepatide versus placebo or semaglutide on pancreatic islet function and insulin sensitivity in adults with type 2 diabetes: a multicentre, randomised, double-blind, parallel-arm, phase 1 clinical trial. Lancet. Diabetes and Endocrinology 10 418–429. (10.1016/S2213-8587(2200085-7) 35468322

[bib82] Hellmér J Marcus C Sonnenfeld T & Arner P 1992 Mechanisms for differences in lipolysis between human subcutaneous and omental fat cells. Journal of Clinical Endocrinology and Metabolism 75 15–20. (10.1210/jcem.75.1.1320047) 1320047

[bib83] Heneghan HM Miller N McAnena OJ O’Brien T & Kerin MJ 2011 Differential miRNA expression in omental adipose tissue and in the circulation of obese patients identifies novel metabolic biomarkers. Journal of Clinical Endocrinology and Metabolism 96 E846–E850. (10.1210/jc.2010-2701) 21367929

[bib84] Hermansen K Bækdal TA Düring M Pietraszek A Mortensen LS Jørgensen H & Flint A 2013 Liraglutide suppresses postprandial triglyceride and apolipoprotein B48 elevations after a fat-rich meal in patients with type 2 diabetes: a randomized, double-blind, placebo-controlled, cross-over trial. Diabetes, Obesity and Metabolism 15 1040–1048. (10.1111/dom.12133) 23683069

[bib85] Herz M Johns D Reviriego J Grossman LD Godin C Duran S Hawkins F Lochnan H Escobar-Jiménez F Hardin PA, *et al.* 2003 A randomized, double-blind, placebo-controlled, clinical trial of the effects of pioglitazone on glycemic control and dyslipidemia in oral antihyperglycemic medication-naive patients with type 2 diabetes mellitus. Clinical Therapeutics 25 1074–1095. (10.1016/s0149-2918(0380068-1) 12809958

[bib86] Home PD Pocock SJ Beck-Nielsen H Curtis PS Gomis R Hanefeld M Jones NP Komajda M McMurray JJV & RECORD Study Team 2009 Rosiglitazone evaluated for cardiovascular outcomes in oral agent combination therapy for type 2 diabetes (RECORD): a multicentre, randomised, open-label trial. Lancet 373 2125–2135. (10.1016/S0140-6736(0960953-3) 19501900

[bib87] Hopper I Billah B Skiba M & Krum H 2011 Prevention of diabetes and reduction in major cardiovascular events in studies of subjects with prediabetes: meta-analysis of randomised controlled clinical trials. European Journal of Cardiovascular Prevention and Rehabilitation 18 813–823. (10.1177/1741826711421687) 21878448

[bib88] Hotamisligil GS 2017 Inflammation, metaflammation and immunometabolic disorders. Nature 542 177–185. (10.1038/nature21363) 28179656

[bib89] Huang-Doran I Zhang CY & Vidal-Puig A 2017 Extracellular vesicles: novel mediators of cell communication in metabolic disease. Trends in Endocrinology and Metabolism 28 3–18. (10.1016/j.tem.2016.10.003) 27810172

[bib240] Ibrahim MM 2010 Subcutaneous and visceral adipose tissue: structural and functional differences. Obesity Reviews 11 11–18. (10.1111/j.1467-789X.2009.00623.x) 19656312

[bib90] Ichimura A Hasegawa S Kasubuchi M & Kimura I 2014 Free fatty acid receptors as therapeutic targets for the treatment of diabetes. Frontiers in Pharmacology 5 1–6. (10.3389/fphar.2014.00236) 25414667 PMC4222138

[bib91] Itoh Y Kawamata Y Harada M Kobayashi M Fujii R Fukusumi S Ogi K Hosoya M Tanaka Y Uejima H, *et al.* 2003 Free fatty acids regulate insulin secretion from pancreatic β cells through GPR40. Nature 422 173–176. (10.1038/nature01478) 12629551

[bib92] Jamaluddin MS Weakley SM Yao Q & Chen C 2012 Resistin: functional roles and therapeutic considerations for cardiovascular disease. British Journal of Pharmacology 165 622–632. (10.1111/j.1476-5381.2011.01369.x) 21545576 PMC3315035

[bib93] Jastreboff AM Aronne LJ Ahmad NN Wharton S Connery L Alves B Kiyosue A Zhang S Liu B Bunck MC, *et al.* 2022 Tirzepatide once weekly for the treatment of obesity. New England Journal of Medicine 387 205–216. (10.1056/NEJMoa2206038) 35658024

[bib95] Kaku K Enya K Nakaya R Ohira T & Matsuno R 2016 Long-term safety and efficacy of fasiglifam (TAK-875), a G-protein-coupled receptor 40 agonist, as monotherapy and combination therapy in Japanese patients with type 2 diabetes: a 52-week open-label phase III study. Diabetes, Obesity and Metabolism 18 925–929. (10.1111/dom.12693) 27178047

[bib96] Karpe F Dickmann JR & Frayn KN 2011 Fatty acids, obesity, and insulin resistance: time for a reevaluation. Diabetes 60 2441–2449. (10.2337/db11-0425) 21948998 PMC3178283

[bib97] Kashyap S Belfort R Gastaldelli A Pratipanawatr T Berria R Pratipanawatr W Bajaj M Mandarino L DeFronzo R & Cusi K 2003 A sustained increase in plasma free fatty acids impairs insulin secretion in nondiabetic subjects genetically predisposed to develop type 2 diabetes. Diabetes 52 2461–2474. (10.2337/diabetes.52.10.2461) 14514628

[bib98] Kebede M Alquier T Latour MG Semache M Tremblay C & Poitout V 2008 The fatty acid receptor GPR40 plays a role in insulin secretion in vivo after high-fat feeding. Diabetes 57 2432–2437. (10.2337/db08-0553) 18559658 PMC2518494

[bib99] Kelly IE Han TS Walsh K & Lean ME 1999 Effects of a thiazolidinedione compound on body fat and fat distribution of patients with type 2 diabetes. Diabetes Care 22 288–293. (10.2337/diacare.22.2.288) 10333947

[bib100] Kersten S 2023 The impact of fasting on adipose tissue metabolism. Biochimica et Biophysica Acta. Molecular and Cell Biology of Lipids 1868 159262. (10.1016/j.bbalip.2022.159262) 36521736

[bib101] Khan S & Kowluru A 2018 CD36 mediates lipid accumulation in pancreatic beta cells under the duress of glucolipotoxic conditions: novel roles of lysine deacetylases. Biochemical and Biophysical Research Communications 495 2221–2226. (10.1016/j.bbrc.2017.12.111) 29274335 PMC5756509

[bib102] Khawandanah J 2019 Double or hybrid diabetes: a systematic review on disease prevalence, characteristics and risk factors. Nutrition and Diabetes 9 33. (10.1038/s41387-019-0101-1) 31685799 PMC6828774

[bib103] Kieffer TJ Heller RS & Habener JF 1996 Leptin receptors expressed on pancreatic beta-cells. Biochemical and Biophysical Research Communications 224 522–527. (10.1006/bbrc.1996.1059) 8702421

[bib104] Kietsiriroje N Pearson S Campbell M Ariëns RAS & Ajjan RA 2019 Double diabetes: a distinct high-risk group? Diabetes, Obesity and Metabolism 21 2609–2618. (10.1111/dom.13848) 31373146

[bib105] Kim JY van de Wall E Laplante M Azzara A Trujillo ME Hofmann SM Schraw T Durand JL Li H Li G, *et al.* 2007 Obesity-associated improvements in metabolic profile through expansion of adipose tissue. Journal of Clinical Investigation 117 2621–2637. (10.1172/JCI31021) 17717599 PMC1950456

[bib106] Klein S Gastaldelli A Yki-Järvinen H & Scherer PE 2022 Why does obesity cause diabetes? Cell Metabolism 34 11–20. (10.1016/j.cmet.2021.12.012) 34986330 PMC8740746

[bib107] Klok MD Jakobsdotti S & Drent ML 2006 The role of leptin and ghrelin in the regulation of food intake and body weight in humans: a review. Obesity Reviews 8 21–34. (10.1111/j.1467-789X.2006.00270.x) 17212793

[bib108] Kratz M Coats BR Hisert KB Hagman D Mutskov V Peris E Schoenfelt KQ Kuzma JN Larson I Billing PS, *et al.* 2014 Metabolic dysfunction drives a mechanistically distinct proinflammatory phenotype in adipose tissue macrophages. Cell Metabolism 20 614–625. (10.1016/j.cmet.2014.08.010) 25242226 PMC4192131

[bib109] Lafontan M & Langin D 2009 Lipolysis and lipid mobilization in human adipose tissue. Progress in Lipid Research 48 275–297. (10.1016/j.plipres.2009.05.001) 19464318

[bib110] Larsson O Deeney JT Bränström R Berggren PO & Corkey BE 1996 Activation of the ATP-sensitive K+ channel by long chain acyl-CoA: a role in modulation of pancreatic β-cell glucose sensitivity. Journal of Biological Chemistry 271 10623–10626. (10.1074/jbc.271.18.10623) 8631866

[bib244] Lee E Korf H & Vidal-Puig A 2023 An adipocentric perspective on the development and progression of non-alcoholic fatty liver disease. Journal of Hepatology 78 1048–1062. (10.1016/j.jhep.2023.01.024) 36740049

[bib112] Li Z Xu X Huang W & Qian H 2018 Free fatty acid receptor 1 (FFAR1) as an emerging therapeutic target for type 2 diabetes mellitus: recent progress and prevailing challenges. Medicinal Research Reviews 38 381–425. (10.1002/med.21441) 28328012

[bib111] Li J Fang J Jiang X Zhang Y Vidal-Puig A & Zhang CY 2024 RNAkines are secreted messengers shaping health and disease. Trends in Endocrinology and Metabolism 35 201–218. (10.1016/j.tem.2023.12.004) 38160178 PMC7617407

[bib113] Liao HW Saver JL Wu YL Chen TH Lee M & Ovbiagele B 2017 Pioglitazone and cardiovascular outcomes in patients with insulin resistance, pre-diabetes and type 2 diabetes: a systematic review and meta-analysis. BMJ Open 7 e013927. (10.1136/bmjopen-2016-013927) PMC5223642 28057658

[bib114] Lindström J Ilanne-Parikka P Peltonen M Aunola S Eriksson JG Hemiö K Hämäläinen H Härkönen P Keinänen-Kiukaanniemi S Laakso M, *et al.* 2006 Sustained reduction in the incidence of type 2 diabetes by lifestyle intervention: follow-up of the finnish diabetes prevention study. Lancet 368 1673–1679. (10.1016/S0140-6736(0669701-8) 17098085

[bib115] Liu J Fox CS Hickson DA May WD Hairston KG Carr JJ & Taylor HA 2010 Impact of abdominal visceral and subcutaneous adipose tissue on cardiometabolic risk factors: the Jackson Heart Study. Journal of Clinical Endocrinology and Metabolism 95 5419–5426. (10.1210/jc.2010-1378) 20843952 PMC2999970

[bib116] Logue J Walker JJ Colhoun HM Leese GP Lindsay RS McKnight JA Morris AD Pearson DW Petrie JR Philip S, *et al.* 2011 Do men develop type 2 diabetes at lower body mass indices than women? Diabetologia 54 3003–3006. (10.1007/s00125-011-2313-3) 21959958 PMC4220585

[bib117] Lu T Wang Y Dou T Xue B Tan Y & Yang J 2019 Pancreatic fat content is associated with β-cell function and insulin resistance in Chinese type 2 diabetes subjects. Endocrine Journal 66 265–270. (10.1507/endocrj.EJ18-0436) 30700664

[bib118] Maeda N Shimomura I Kishida K Nishizawa H Matsuda M Nagaretani H Furuyama N Kondo H Takahashi M Arita Y, *et al.* 2002 Diet-induced insulin resistance in mice lacking adiponectin/ACRP30. Nature Medicine 8 731–737. (10.1038/nm724) 12068289

[bib238] Maedler K Oberholzer J Bucher P Spinas GA & Donath MY 2023 Monounsaturated fatty acids prevent the deleterious effects of palmitate and high glucose on human pancreatic beta-cell turnover and function. Diabetes 52 726–733. (10.2337/diabetes.52.3.726) 12606514

[bib242] Mancuso P 2016 The role of adipokines in chronic inflammation. Immunotargets and Therapy 5 47–56. (10.2147/itt.s73223) 27529061 PMC4970637

[bib119] March CA Becker DJ & Libman IM 2021 Nutrition and obesity in the pathogenesis of youth-onset type 1 diabetes and its complications. Frontiers in Endocrinology (Lausanne) 12 1–15. 10.3389/fendo.2021.622901 PMC8021094 33828529

[bib120] Mårin P Andersson B Ottosson M Olpe L Chowdhury B Kvist H Holm G Sjostrom L & Bjorntorp P 1992 The morphology and metabolism of intraabdominal adipose tissue in men. Metabolism: Clinical and Experimental 41 1242–1248. (10.1016/0026-0495(9290016-4) 1435298

[bib121] Marselli L Piron A Suleiman M Colli ML Yi X Khamis A Carrat GR Rutter GA Bugliani M Giusti L, *et al.* 2020 Persistent or transient human β cell dysfunction induced by metabolic stress: specific signatures and shared gene expression with type 2 diabetes. Cell Reports 33 108466. (10.1016/j.celrep.2020.108466) 33264613

[bib122] Mathieu C Zinman B Hemmingsson JU Woo V Colman P Christiansen E Linder M Bode B & ADJUNCT ONE Investigators 2016 Efficacy and safety of liraglutide added to insulin treatment in type 1 diabetes: the ADJUNCT ONE treat-to-target randomized trial. Diabetes Care 39 1702–1710. (10.2337/dc16-0691) 27506222

[bib123] Matsushima-Nagata K Matsumura T Kondo Y Anraku K Fukuda K Yamanaka M Manabe M Irie T Araki E & Sugiuchi H 2023 Significance of circulating remnant lipoprotein cholesterol levels measured by homogeneous assay in patients with type 2 diabetes. Biomolecules 13. (10.3390/biom13030468) PMC10099722 36979403

[bib124] McGarry JD 2002 Banting lecture 2001: Dysregulation of fatty acid metabolism in the etiology of type 2 diabetes. Diabetes 51 7–18. (10.2337/diabetes.51.1.7) 11756317

[bib126] McLaughlin TM Liu T Yee G Abbasi F Lamendola C Reaven GM Tsao P Cushman SW & Sherman A 2010 Pioglitazone increases the proportion of small cells in human abdominal subcutaneous adipose tissue. Obesity 18 926–931. (10.1038/oby.2009.380) 19910937 PMC9413023

[bib125] McLaughlin T Lamendola C Liu A & Abbasi F 2011 Preferential fat deposition in subcutaneous versus visceral depots is associated with insulin sensitivity. Journal of Clinical Endocrinology and Metabolism 96 E1756–E1760. (10.1210/jc.2011-0615) 21865361 PMC3205890

[bib127] McQuaid SE Hodson L Neville MJ Dennis AL Cheeseman J Humphreys SM Ruge T Gilbert M Fielding BA Frayn KN, *et al.* 2011 Downregulation of adipose tissue fatty acid trafficking in obesity: a driver for ectopic fat deposition? Diabetes 60 47–55. (10.2337/db10-0867) 20943748 PMC3012196

[bib128] Medina-Gomez G Gray SL Yetukuri L Shimomura K Virtue S Campbell M Curtis RK, Jimenez-Linan M Blount M Yeo GSH, *et al.* 2007 PPAR gamma 2 prevents lipotoxicity by controlling adipose tissue expandability and peripheral lipid metabolism. PLoS Genetics 3 0634–0647. 10.1371/journal.pgen.0030064 PMC1857730 17465682

[bib129] Menzaghi C Coco A Salvemini L Thompson R De Cosmo S Doria A & Trischitta V 2006 Heritability of serum resistin and its genetic correlation with insulin resistance-related features in nondiabetic Caucasians. Journal of Clinical Endocrinology and Metabolism 91 2792–2795. (10.1210/jc.2005-2715) 16670163

[bib130] Merger SR Kerner W Stadler M Zeyfang A Jehle P Müller-Korbsch M Holl RW, DPV Initiative & German BMBF Competence Network Diabetes mellitus 2016 Prevalence and comorbidities of double diabetes. Diabetes Research and Clinical Practice 119 48–56. (10.1016/j.diabres.2016.06.003) 27449710

[bib131] Miles JM Wooldridge D Grellner WJ Windsor S Isley WL Klein S & Harris WS 2003 Nocturnal and postprandial free fatty acid kinetics in normal and type 2 diabetic subjects: effects of insulin sensitization therapy. Diabetes 52 675–681. (10.2337/diabetes.52.3.675) 12606508

[bib134] Misra A & Vikram NK 2003 Clinical and pathophysiological consequences of abdominal adiposity and abdominal adipose tissue depots. Nutrition 19 457–466. (10.1016/s0899-9007(0201003-1) 12714101

[bib132] Mishra BK Shukla P Aslam M Siddiqui AA & Madhu SV 2018 Prevalence of double diabetes in youth onset diabetes patients from east Delhi and neighboring NCR region. Diabetes and Metabolic Syndrome 12 839–842. (10.1016/j.dsx.2017.08.016) 28899639

[bib133] Mishra SP Karunakar P Taraphder S & Yadav H 2020 Free fatty acid receptors 2 and 3 as microbial metabolite sensors to shape host health: pharmacophysiological view. Biomedicines 8. (10.3390/biomedicines8060154) PMC7344995 32521775

[bib135] Miyazaki Y Mahankali A Matsuda M Glass L Mahankali S Ferrannini E Cusi K Mandarino LJ & DeFronzo RA, Mandarin 2001 Improved glycemic control and enhanced insulin sensitivity in type 2 diabetic subjects treated with pioglitazone. Diabetes Care 24 710–719. (10.2337/diacare.24.4.710) 11315836

[bib136] Moffitt JH Fielding BA Evershed R Berstan R Currie JM & Clark A 2005 Adverse physicochemical properties of tripalmitin in beta cells lead to morphological changes and lipotoxicity in vitro. Diabetologia 48 1819–1829. (10.1007/s00125-005-1861-9) 16094531

[bib137] Mori Y Murakawa Y Okada K Horikoshi H Yokoyama J Tajima N & Ikeda Y 1999 Effect of troglitazone on body fat distribution in type 2 diabetic patients. Diabetes Care 22 908–912. (10.2337/diacare.22.6.908) 10372240

[bib138] Morigny P Boucher J Arner P & Langin D 2021 Lipid and glucose metabolism in white adipocytes: pathways, dysfunction and therapeutics. Nature Reviews. Endocrinology 17 276–295. (10.1038/s41574-021-00471-8) 33627836

[bib139] Morrison AE Zaccardi F Khunti K & Davies MJ 2019 Causality between non-alcoholic fatty liver disease and risk of cardiovascular disease and type 2 diabetes: a meta-analysis with bias analysis. Liver International 39 557–567. (10.1111/liv.13994) 30358050

[bib140] Mugabo Y Zhao S Lamontagne J Al-Mass A Peyot ML Corkey BE Joly E Madiraju SPM & Prentki M 2017 Metabolic fate of glucose and candidate signaling and excess-fuel detoxification pathways in pancreatic β-cells. Journal of Biological Chemistry 292: 7407–7422. 28280244 10.1074/jbc.M116.763060 PMC5418042

[bib141] Mziaut H Henniger G Ganss K Hempel S Wolk S McChord J Chowdhury K Ravassard P Knoch KP Krautz C, *et al.* 2020 MiR-132 controls pancreatic beta cell proliferation and survival through Pten/Akt/Foxo3 signaling. Molecular Metabolism 31 150–162. (10.1016/j.molmet.2019.11.012) 31918917 PMC6928290

[bib142] Nagao M Esguerra JLS Asai A Ofori JK Edlund A Wendt A Sugihara H Wollheime CB Oikawa S & Eliasson L 2020 Potential protection against type 2 diabetes in obesity through lower CD36 expression and improved exocytosis in β-cells. Diabetes 69 1193–1205. (10.2337/db19-0944) 32198214 PMC7243297

[bib143] Nakata M Okada T Ozawa K & Yada T 2007 Resistin induces insulin resistance in pancreatic islets to impair glucose-induced insulin release. Biochemical and Biophysical Research Communications 353 1046–1051. (10.1016/j.bbrc.2006.12.134) 17207771

[bib144] Natali A Gastaldelli A Camastra S Baldi S Quagliarini F Minicocci I Bruno C Pennisi E & Arca M 2013 Metabolic consequences of adipose triglyceride lipase deficiency in humans: an in vivo study in patients with neutral lipid storage disease with myopathy. Journal of Clinical Endocrinology and Metabolism 98 E1540–E1548. (10.1210/jc.2013-1444) 23824421

[bib236] Nettebrock NT & Bohnert M 2020 Born this way - biogenesis of lipid droplets from specialized ER subdomains. Biochimica et Biophysica Acta - Molecular and Cell Biology of Lipids 1865 158448. (10.1016/j.bbalip.2019.04.008) 31028912

[bib146] Nielsen S Guo ZK Johnson CM Hensrud DD & Jensen MD 2004 Splanchnic lipolysis in human obesity. Journal of Clinical Investigation 113 1582–1588. (10.1172/JCI21047) 15173884 PMC419492

[bib147] Nissen SE & Wolski K 2007 Effect of rosiglitazone on the risk of myocardial infarction and death from cardiovascular causes. New England Journal of Medicine 356 2457–2471. (10.1056/NEJMoa072761) 17517853

[bib148] Nolan CJ Leahy JL Delghingaro-Augusto V Moibi J Soni K Peyot ML Fortier M Guay C Lamontagne J Barbeau A, *et al.* 2006 Beta cell compensation for insulin resistance in Zucker fatty rats: increased lipolysis and fatty acid signalling. Diabetologia 49 2120–2130. (10.1007/s00125-006-0305-5) 16868750

[bib149] Noushmehr H D’Amico E Farilla L Hui H Wawrowsky KA Mlynarski W Doria A Abumrad NA & Perfetti R 2005 Fatty acid translocase (FAT/CD36) is localized on insulin-containing granules in human pancreatic β-cells and mediates fatty acid effects on insulin secretion. Diabetes 54 472–481. (10.2337/diabetes.54.2.472) 15677505

[bib150] Ogilvie RF 1933 The islands of langerhans in 19 cases of obesity. Journal of Pathology and Bacteriology 37 473–481. (10.1002/path.1700370314)

[bib151] Oh YS Bae GD Baek DJ Park EY & Jun HS 2018 Fatty acid-induced lipotoxicity in pancreatic beta-cells during development of type 2 diabetes. Frontiers in Endocrinology (Lausanne) 9 1–10) 10.3389/fendo.2018.00384 PMC6054968 30061862

[bib152] Ohneda A Aguilar-Parada E Eisentraut AM & Unger RH 1969 Control of pancreatic glucagon secretion by glucose. Diabetes 18 1–10. (10.2337/diab.18.1.1) 5761864

[bib153] Olofsson CS Salehi A Göpel SO Holm C & Rorsman P 2004 Palmitate stimulation of glucagon secretion in mouse pancreatic alpha-cells results from activation of L-type calcium channels and elevation of cytoplasmic calcium. Diabetes 53 2836–2843. (10.2337/diabetes.53.11.2836) 15504963

[bib154] Olsen TS 1978 Lipomatosis of the pancreas in autopsy material and its relation to age and overweight. Acta Pathologica et Microbiologica Scandinavica. Section A, Pathology 86A 367–373. (10.1111/j.1699-0463.1978.tb02058.x) 716899

[bib155] Pan X Kaminga AC Wen SW Acheampong K & Liu A 2019 Omentin-1 in diabetes mellitus: a systematic review and meta-analysis. PLoS One 14 1–17. (10.1371/journal.pone.0226292) PMC6903756 31821362

[bib156] Petrie JR Chaturvedi N Ford I Brouwers MCGJ Greenlaw N Tillin T Hramiak I Hughes AD Jenkins AJ Klein BEK, *et al.* 2017 Cardiovascular and metabolic effects of metformin in patients with type 1 diabetes (REMOVAL): a double-blind, randomised, placebo-controlled trial. Lancet. Diabetes and Endocrinology 5 597–609. (10.1016/S2213-8587(1730194-8) 28615149 PMC5641446

[bib157] Panzer JK & Caicedo A 2021 Targeting the pancreatic a-cell to prevent hypoglycemia in type 1 diabetes. Diabetes 70 2721–2732. (10.2337/dbi20-0048) 34872936 PMC8660986

[bib158] Paolisso G Tagliamonte MR Rizzo MR Gualdiero P Saccomanno F Gambardella A Giugliano D D’Onofrio F & Howard BV 1998 Lowering fatty acids potentiates acute insulin response in first degree relatives of people with type II diabetes. Diabetologia 41 1127–1132. (10.1007/s001250051041) 9794097

[bib159] Pappan KL Pan Z Kwon G Marshall CA Coleman T Goldberg IJ McDaniel ML & Semenkovich CF 2005 Pancreatic β-cell lipoprotein lipase independently regulates islet glucose metabolism and normal insulin secretion. Journal of Biological Chemistry 280 9023–9029. (10.1074/jbc.M409706200) 15637076

[bib160] Petrus P Edholm D Rosqvist F Dahlman I Sundbom M Arner P Ryden M & Riserus U 2017 Depot-specific differences in fatty acid composition and distinct associations with lipogenic gene expression in abdominal adipose tissue of obese women. International Journal of Obesity 41 1295–1298. (10.1038/ijo.2017.106) 28465608 PMC5550557

[bib161] Pichè M-E Parry SA Karpe F & Hodson L 2018 Chylomicron-derived fatty acid spillover in adipose tissue: a signature of metabolic health? Journal of Clinical Endocrinology and Metabolism 103 25–34. (10.1210/jc.2017-01517) 29099975 PMC5761493

[bib162] Pinnick KE Collins SC Londos C Gauguier D Clark A & Fielding BA 2008 Pancreatic ectopic fat is characterized by adipocyte infiltration and altered lipid composition. Obesity (Silver Spring) 16 522–530. (10.1038/oby.2007.110) 18239594

[bib163] Piro S Maniscalchi ET Monello A Pandini G Mascali LG Rabuazzo AM & Purrello F 2010 Palmitate affects insulin receptor phosphorylation and intracellular insulin signal in a pancreatic alpha-cell line. Endocrinology 151 4197–4206. (10.1210/en.2009-1472) 20573722

[bib164] Pownall H & Moore K 2014 Commentary on fatty acid wars: the diffusionists versus the translocatists. Arteriosclerosis, Thrombosis, and Vascular Biology 34 e8–e9. (10.1161/ATVBAHA.114.303380) 24651680 PMC4029155

[bib165] Prager R Wallace P & Olefsky JM 1987 Direct and indirect effects of insulin to inhibit hepatic glucose output in obese subjects. Diabetes 36 607–611. (10.2337/diab.36.5.607) 3552793

[bib167] Prentki M & Madiraju SRM 2012 Glycerolipid/free fatty acid cycle and islet β-cell function in health, obesity and diabetes. Molecular and Cellular Endocrinology 353 88–100. (10.1016/j.mce.2011.11.004) 22108437

[bib166] Prentki M Joly E El-Assaad W & Roduit R 2002 Malonyl-CoA signaling, lipid partitioning, and glucolipotoxicity: role in beta-cell adaptation and failure in the etiology of diabetes. Diabetes 51(Supplement 3) S405–S413. (10.2337/diabetes.51.2007.s405) 12475783

[bib168] Prentki M Matschinsky FM & Madiraju SRM 2013 Metabolic signaling in fuel-induced insulin secretion. Cell Metabolism 18 162–185. (10.1016/j.cmet.2013.05.018) 23791483

[bib170] Quiclet C Dittberner N Gässler A Stadion M Gerst F Helms A Baumeier C Schulz TJ & Schürmann A 2019 Pancreatic adipocytes mediate hypersecretion of insulin in diabetes-susceptible mice. Metabolism: Clinical and Experimental 97 9–17. (10.1016/j.metabol.2019.05.005) 31108105

[bib171] Qureshi W Santaren ID Hanley AJ Watkins SM Lorenzo C & Wagenknecht LE 2019 Risk of diabetes associated with fatty acids in the de novo lipogenesis pathway is independent of insulin sensitivity and response: the Insulin Resistance Atherosclerosis Study (IRAS). BMJ Open Diabetes Research and Care 7 e000691. (10.1136/bmjdrc-2019-000691) PMC6731783 31543975

[bib172] Ratner RE Dickey R Fineman M Maggs DG Shen L Strobel SA Weyer C & Kolterman OG 2004 Amylin replacement with pramlintide as an adjunct to insulin therapy improves long-term glycaemic and weight control in type 1 diabetes mellitus: a 1-year, randomised controlled trial. Diabetic Medicine 21 1204–1212. (10.1111/j.1464-5491.2004.01319.x) 15498087

[bib174] Roduit R Nolan C Alarcon C Moore P Barbeau A Delghingaro-Augusto V Przybykowski E Morin J Massé F Massie B, *et al.* 2004 A role for the malonyl-CoA/long-chain acyl-CoA pathway of lipid signaling in the regulation of insulin secretion in response to both fuel and nonfuel stimuli. Diabetes 53 1007–1019. (10.2337/diabetes.53.4.1007) 15047616

[bib176] Roust LR & Jensen MD 1993 Postprandial free fatty acid kinetics are abnormal in upper body obesity. Diabetes 42 1567–1573. (10.2337/diab.42.11.1567) 8405696

[bib177] Russo L & Lumeng CN 2018 Properties and functions of adipose tissue macrophages in obesity. Immunology 155 407–417. (10.1111/imm.13002) 30229891 PMC6230999

[bib178] Ryan AS & Li G 2022 Adipose and skeletal muscle expression of adiponectin and liver receptor homolog-1 with weight loss and aerobic exercise. Journal of the Endocrine Society 6 bvac095. (10.1210/jendso/bvac095) 35854979 PMC9281870

[bib179] Sadur CN & Eckel RH 1982 Insulin stimulation of adipose tissue lipoprotein lipase. Use of the euglycemic clamp technique. Journal of Clinical Investigation 69 1119–1125. (10.1172/jci110547) 7040473 PMC370176

[bib180] Sassek M Pruszynska-Oszmalek E Kołodziejski PA Szczepankiewicz D Kaczmarek P Wieloch M Kurto K Nogowski L Nowak KW Strowski MZ, *et al.* 2016 Resistin is produced by rat pancreatic islets and regulates insulin and glucagon in vitro secretion. Islets 8 177–185. (10.1080/19382014.2016.1251538) 27797297 PMC5161143

[bib181] Sato S Imachi H Lyu J Miyai Y Fukunaga K Dong T Ibata T Kobayashi T Yoshimoto T Kikuchi F, *et al.* 2018 Effect of TNF-α on the expression of ABCA1 in pancreatic Β-cells. Journal of Molecular Endocrinology 61 185–193. (10.1530/JME-18-0167) 30131353

[bib182] Semple RK Chatterjee VKK & O'Rahilly S 2006 PPARγ and human metabolic disease. Journal of Clinical Investigation 116 581–589. (10.1172/JCI28003) 16511590 PMC1386124

[bib183] Seufert J Kieffer TJ & Habener JF 1999 Leptin inhibits insulin gene transcription and reverses hyperinsulinemia in leptin-deficient ob/ob mice. PNAS 96 674–679. (10.1073/pnas.96.2.674) 9892692 PMC15195

[bib184] Shadid S & Jensen MD 2003 Effects of pioglitazone versus diet and exercise on metabolic health and fat distribution in upper body obesity. Diabetes Care 26 3148–3152. (10.2337/diacare.26.11.3148) 14578253

[bib185] Shimabukuro M Higa M Zhou YT Wang MY Newgard CB & Unger RH 1998a Lipoapoptosis in beta-cells of obese prediabetic fa/fa rats. Role of serine palmitoyltransferase overexpression. Journal of Biological Chemistry 273 32487–32490. (10.1074/jbc.273.49.32487) 9829981

[bib186] Shimabukuro M Zhou YT Levi M & Unger RH 1998b Fatty acid-induced β cell apoptosis: a link between obesity and diabetes. PNAS 95 2498–2502. (10.1073/pnas.95.5.2498) 9482914 PMC19389

[bib187] Shrestha N Reinert RB & Qi L 2020 Endoplasmic reticulum protein quality control in β cells. Seminars in Cell and Developmental Biology 103 59–67. (10.1016/j.semcdb.2020.04.006) 32402517 PMC7321887

[bib188] Sims EAH 2001 Are there persons who are obese, but metabolically healthy? Metabolism: Clinical and Experimental 50 1499–1504. (10.1053/meta.2001.27213) 11735101

[bib189] Skurk T Ecklebe S & Hauner H 2007 A novel technique to propagate primary human preadipocytes without loss of differentiation capacity. Obesity (Silver Spring) 15 2925–2931. (10.1038/oby.2007.349) 18198300

[bib190] Smits MM & Van Geenen EJM 2011 The clinical significance of pancreatic steatosis. Nature Reviews. Gastroenterology and Hepatology 8 169–177. (10.1038/nrgastro.2011.4) 21304475

[bib192] Sniderman AD 2000 Postprandial hypertriglyceridemia(s): time to enlarge our pathophysiologic perspective. European Journal of Clinical Investigation 30 935–937. (10.1046/j.1365-2362.2000.00733.x) 11114954

[bib193] Stein DT Esser V Stevenson BE Lane KE Whiteside JH Daniels MB Chen S & McGarry JD 1996 Essentiality of circulating fatty acids for glucose-stimulated insulin secretion in the fasted rat. Journal of Clinical Investigation 97 2728–2735. (10.1172/JCI118727) 8675683 PMC507365

[bib194] Stein DT Stevenson BE Chester MW Basit M Daniels MB Turley SD & McGarry JD 1997 The insulinotropic potency of fatty acids is influenced profoundly by their chain length and degree of saturation. Journal of Clinical Investigation 100 398–403. (10.1172/JCI119546) 9218517 PMC508203

[bib195] Su KZ Li YR Zhang D Yuan JH Zhang CS Liu Y Song LM Lin Q Li MW & Dong J 2019 Relation of circulating resistin to insulin resistance in type 2 diabetes and obesity: a systematic review and meta-analysis. Frontiers in Physiology 10 1399. (10.3389/fphys.2019.01399) 31803062 PMC6877503

[bib196] Sztalryd C & Brasaemle DL 2017 The perilipin family of lipid droplet proteins: gatekeepers of intracellular lipolysis. Biochimica et Biophysica Acta. Molecular and Cell Biology of Lipids 1862 1221–1232. (10.1016/j.bbalip.2017.07.009) 28754637 PMC5595658

[bib243] Tanabe K Okuya S Tanizawa Y Matsutani A & Oka Y 1997 Leptin induces proliferation of pancreatic beta cell line MIN6 through activation of mitogen-activated protein kinase. Biochemical and Biophysical Research Communications 241 765–768. (10.1006/bbrc.1997.7894) 9434783

[bib198] Tang T Abbott MJ Ahmadian M Lopes AB Wang Y & Sul HS 2013 Desnutrin/ATGL activates PPARδ to promote mitochondrial function for insulin secretion in islet β cells. Cell Metabolism 18 883–895. (10.1016/j.cmet.2013.10.012) 24268737 PMC3871209

[bib197] Tang S Luo F Feng YM Wei X Miao H Lu YB Tang Y Ding DF Jin JF & Zhu Q 2017 Neutral ceramidase secreted via exosome protects against palmitate-induced apoptosis in INS-1 cells. Experimental and Clinical Endocrinology and Diabetes 125 130–135. (10.1055/s-0042-116314) 28008587

[bib199] Targher G Corey KE Byrne CD & Roden M 2021 The complex link between NAFLD and type 2 diabetes mellitus — mechanisms and treatments. Nature Reviews. Gastroenterology and Hepatology 18 599–612. (10.1038/s41575-021-00448-y) 33972770

[bib200] Taylor R Al-Mrabeh A Zhyzhneuskaya S Peters C Barnes AC Aribisala BS Hollingwsworth KG Mathers JC Sattar N & Lean MEJ 2018 Remission of human type 2 diabetes requires decrease in liver and pancreas fat content but is dependent upon capacity for β cell recovery. Cell Metabolism 28 547–556.e3. 30078554 10.1016/j.cmet.2018.07.003

[bib202] Tong X Dai C Walker JT Nair GG Kennedy A Carr RM Hebrok M Powers AC & Stein R 2020 Lipid droplet accumulation in human pancreatic islets is dependent on both donor age and health. Diabetes 69 342–354. (10.2337/db19-0281) 31836690 PMC7034188

[bib203] Trevino MB Machida Y Hallinger DR Garcia E Christensen A Dutta S Peake DA Ikeda Y & Imai Y 2015 Perilipin 5 regulates islet lipid metabolism and insulin secretion in a cAMP-dependent manner: implication of its role in the postprandial insulin secretion. Diabetes 64 1299–1310. (10.2337/db14-0559) 25392244 PMC4375085

[bib204] Tushuizen ME Bunck MC Pouwels PJ Bontemps S Van Waesberghe JHT Schindhelm RK Mari A Heine RJ & Diamant M 2007 Pancreatic fat content and β-cell function in men with and without type 2 diabetes. Diabetes Care 30 2916–2921. (10.2337/dc07-0326) 17666465

[bib201] U. S. Food and Drug Administration (FDA) 2015 FDA Drug Safety Communication: FDA Eliminates the Risk Evaluation and Mitigation Strategy (REMS) for Rosiglitazone-Containing Diabetes Medicines. Silver Spring, MD, USA: FDA. (available at: https://www.fda.gov/drugs/drug-safety-and-availability/fda-drug-safety-communication-fda-eliminates-risk-evaluation-and-mitigation-strategy-rems)

[bib205] Vague J 1947 Sexual differentiation. A determinant factor of the forms of obesity. La Presse Médicale 30 339–340. 18918084

[bib206] Van der Schueren B Ellis D Faradji RN Al-Ozairi E Rosen J & Mathieu C 2021 Obesity in people living with type 1 diabetes. Lancet. Diabetes and Endocrinology 9 776–785. (10.1016/S2213-8587(2100246-1) 34600607

[bib207] Van Der Zijl NJ Goossens GH Moors CCM Van Raalte DH Muskiet MHA Pouwels PJW Blaak EE & Diamant M 2011 Ectopic fat storage in the pancreas, liver, and abdominal fat depots: impact on β-cell function in individuals with impaired glucose metabolism. Journal of Clinical Endocrinology and Metabolism 96 459–467. (10.1210/jc.2010-1722) 21084401

[bib208] Van Harmelen V Reynisdottir S Eriksson P Thorne A Hoffstedt J Lonnqvist F & Arner P 1998 Leptin secretion from subcutaneous and visceral adipose tissue in women. Diabetes 47 913–917. (10.2337/diabetes.47.6.913) 9604868

[bib209] Vatier C Bidault G Briand N Guénantin A-C Teyssières L Lascols O Capeau J & Vigouroux C 2013 What the genetics of lipodystrophy can teach us about insulin resistance and diabetes. Current Diabetes Reports 13 757–767. (10.1007/s11892-013-0431-7) 24026869

[bib210] Vatier C Fetita S Boudou P Tchankou C Deville L Riveline J Young J Mathivon L Travert F Morin D, *et al.* 2016 One-year Metreleptin improves insulin secretion in patients with diabetes linked to genetic lipodystrophic syndromes. Diabetes, Obesity and Metabolism 18 693–697. (10.1111/dom.12606) 26584826

[bib211] Verbeeten KC Elks CE Daneman D & Ong KK 2011 Association between childhood obesity and subsequent type 1 diabetes: a systematic review and meta-analysis. Diabetic Medicine 28 10–18. (10.1111/j.1464-5491.2010.03160.x) 21166841

[bib212] Véret J Coant N Berdyshev EV Skobeleva A Therville N Bailbé D Gorshkova I Natarajan V Portha B & Le Stunff H 2011 Ceramide synthase 4 and de novo production of ceramides with specific N-acyl chain lengths are involved in glucolipotoxicity-induced apoptosis of INS-1 β-cells. Biochemical Journal 438 177–189. (10.1042/BJ20101386) 21592087

[bib213] Véret J Coant N Gorshkova IA Giussani P Fradet M Riccitelli E Skobeleva A Goya J Kassis N Natarajan V, *et al.* 2013 Role of palmitate-induced sphingoid base-1-phosphate biosynthesis in INS-1 β-cell survival. Biochimica et Biophysica Acta 1831 251–262. (10.1016/j.bbalip.2012.10.003) 23085009

[bib214] Vernier S Chiu A Schober J Weber T Nguyen P Luer M McPherson T Wanda PE Marshall CA Rohatgi N, *et al.* 2012 Β-cell metabolic alterations under chronic nutrient overload in rat and human islets. Islets 4 379–392. (10.4161/isl.22720) 23247575 PMC3605166

[bib215] Villaret A Galitzky J Decaunes P Estève D Marques MA Sengenès C Chiotasso P Tchkonia T Lafontan M Kirkland JL, *et al.* 2010 Adipose tissue endothelial cells from obese human subjects: differences among depots in angiogenic, metabolic, and inflammatory gene expression and cellular senescence. Diabetes 59 2755–2763. (10.2337/db10-0398) 20713685 PMC2963533

[bib216] Virtue S & Vidal-Puig A 2010 Adipose tissue expandability, lipotoxicity and the metabolic syndrome - an allostatic perspective. Biochimica et Biophysica Acta 1801 338–349. (10.1016/j.bbalip.2009.12.006) 20056169

[bib217] Voukali M Kastrinelli I Stragalinou S Tasiopoulou D Paraskevopoulou P Katsilambros N Kokkinos A Tentolouris N & Ioannidis I 2014 Study of postprandial lipaemia in type 2 diabetes mellitus: exenatide versus liraglutide. Journal of Diabetes Research 2014 304032. (10.1155/2014/304032) PMC4137738 25165723

[bib219] Wagner R Eckstein SS Yamazaki H Gerst F Machann J Jaghutriz BA Schurmann A Solimena M Singer S Konigsrainer A, *et al.* 2022 Metabolic implications of pancreatic fat accumulation. Nature Reviews. Endocrinology 18 43–54. (10.1038/s41574-021-00573-3) 34671102

[bib220] Wang L Folsom AR Zheng Z-J Pankow JS Eckfeldt JH & ARIC Study Investigators 2003 Plasma fatty acid composition and incidence of diabetes in middle-aged adults: the Atherosclerosis Risk in Communities (ARIC) Study. American Journal of Clinical Nutrition 78 91–98. (10.1093/ajcn/78.1.91) 12816776

[bib221] Wang L Zhao Y Gui B Fu R Ma F Yu J Qu P Dong L & Chen C 2011 Acute stimulation of glucagon secretion by linoleic acid results from GPR40 activation and [Ca^2+^]_i_ increase in pancreatic islet α-cells. Journal of Endocrinology 210 173–179. (10.1530/JOE-11-0132) 21565851

[bib218] Wang JY Wang QW Yang XY Yang W Li DR Jin JY Zhang HC & Zhang X 2023 FGLP-1 receptor agonists for the treatment of obesity: role as a promising approach. Frontiers in Endocrinology (Lausanne) 14 1–11. 10.3389/fendo.2023.1085799 PMC9945324 36843578

[bib222] Welte MA & Gould AP 2017 Lipid droplet functions beyond energy storage. Biochimica et Biophysica Acta. Molecular and Cell Biology of Lipids 1862 1260–1272. (10.1016/j.bbalip.2017.07.006) 28735096 PMC5595650

[bib223] White U Fitch MD Beyl RA Hellerstein MK & Ravussin E 2021 Adipose depot-specific effects of 16 weeks of pioglitazone on in vivo adipogenesis in women with obesity: a randomised controlled trial. Diabetologia 64 159–167. (10.1007/s00125-020-05281-7) 33001232 PMC7718382

[bib224] Wilkin TJ 2009 The accelerator hypothesis: a review of the evidence for insulin resistance as the basis for type I as well as type II diabetes. International Journal of Obesity 33 716–726. (10.1038/ijo.2009.97) 19506563

[bib225] Wing RR & Cleary PA 1988 Weight gain associated with intensive therapy in the diabetes control and complications trial. Diabetes Care 11 567–573. (10.2337/diacare.11.7.567) 2904881

[bib227] Yamazaki H Tauchi S Machann J Haueise T Yamamoto Y Dohke M Hanawa N Kodama Y Katanuma A Stefan N, *et al.* 2022 Fat distribution patterns and future type 2 diabetes. Diabetes 71 1937–1945. (10.2337/db22-0315) 35724270

[bib228] Yan MX Li YQ Meng M Ren HB & Kou Y 2006 Long-term high-fat diet induces pancreatic injuries via pancreatic microcirculatory disturbances and oxidative stress in rats with hyperlipidemia. Biochemical and Biophysical Research Communications 347 192–199. (10.1016/j.bbrc.2006.06.063) 16814251

[bib229] Yaney GC Korchak HM & Corkey BE 2000 Long-chain acyl CoA regulation of protein kinase C and fatty acid potentiation of glucose-stimulated insulin secretion in clonal β-cells. Endocrinology 141 1989–1998. (10.1210/endo.141.6.7493) 10830281

[bib230] Yang Q Loureiro ZY Desai A SeSouza T Li K Wang H Nicoloro SM Solivan-Rivera J & Corvera S 2023 Regulation of lipolysis by 14–3-3 proteins on human adipocyte lipid droplets. PNAS Nexus 2 pgad420. (10.1093/pnasnexus/pgad420) 38130664 PMC10733194

[bib231] Ye R Wang M Wang QA & Scherer PE 2015 Adiponectin-mediated antilipotoxic effects in regenerating pancreatic islets. Endocrinology 156 2019–2028. (10.1210/en.2015-1066) 25815422 PMC4430619

[bib232] Yew Tan C Virtue S Murfitt S Roberts LD Phua YH Dale M Griffin JL Tinajones F Scherer PE & Vidal-Puig A 2016 Adipose tissue fatty acid chain length and mono-unsaturation increases with obesity and insulin resistance. Scientific Reports 5 18366. (10.1038/srep18366). [Erratum: Scientific Reports 6 23873. (10.1038/srep18366)] PMC4683622 26679101

[bib233] Yip RGC & Wolfe MM 1999 GIP biology and fat metabolism. Life Sciences 66 91–103. (10.1016/s0024-3205(9900314-8) 10666005

[bib234] Zhang S & Kim KH 1995 TNF-alpha inhibits glucose-induced insulin secretion in a pancreatic beta-cell line (INS-1). FEBS Letters 377 237–239. (10.1016/0014-5793(9501272-9) 8543058

[bib235] Zhang Y Proenca R Maffei M Barone M Leopold L & Friedman JM 1994 Positional cloning of the mouse obese gene and its human homologue. Nature 372 425–432. (10.1038/372425a0) 7984236

